# Supplementary material for: Validity of the International Fitness Scale (IFIS) and its associations with cardiometabolic health and body composition in adults with type 2 diabetes: A cross-sectional study
Source: PLoS One. 2026 Jan 6;21(1):e0339364. doi: 10.1371/journal.pone.0339364 (PMC12774367; doi:10.1371/journal.pone.0339364)
Supplement: Appendix 1 — (DOCX) [file pone.0339364.s001.docx]

**Calculation of the Life’s Essential 8 Factor score**

In consonance with the American Heart Association, the Life’s Essential 8 Factor (LE8 Factor) score, was derived from four factors closely associated with cardiovascular health: body mass index (BMI), blood lipids, blood pressure, and diabetes.[1]

**Body mass index**

Height and weight were measured with participants wearing light indoor clothing, and body mass index (BMI) was calculated using the standard formula (weight in kilograms divided by height in meters squared, kg/m²).

| **BMI** | **Score** |
| --- | --- |
| <25.0 | 100 |
| 25.0-29.9 | 70 |
| 30.0-34.9 | 30 |
| 35.0-39.9 | 15 |
| ≥40.0 | 0 |

**Blood lipids**

Venous blood samples for lipid analysis were collected after an overnight fast of at least 6 hours.

| **Non high density lipoprotein (HDL) cholesterol (mg/dl)** | **Score** |
| --- | --- |
| <130.0 | 100 |
| 130.0-159.9 | 60 |
| 160.0-189.9 | 40 |
| 190.0-219.9 | 20 |
| ≥220.0 | 0 |
| Non-HDL cholesterol was calculated as total cholesterol – HDL cholesterol.  If drug-treated level, 20 points were subtracted. | |

**Blood pressure**

Blood pressure (mmHg) was measured after the participant had been resting in a supine position for at least five minutes.

| **Systolic blood pressure (SBP) and diastolic blood pressure (DBP) (mmHg)** | **Score** |
| --- | --- |
| <120.0/<80.0 | 100 |
| 120.0–129.9/<80.0 | 75 |
| 130.0–139.9 or 80.0–89.9 | 50 |
| 140.0–159.9 or 90.0–99.9 | 25 |
| ≥160.0 or ≥100.0 | 0 |
| *The worst scenario is considered, e.g., a patient with SBP=135.0 and DBP=95.0 scores 25 points.  If drug-treated level 20 points were subtracted. | |

**Blood glucose**

Venous blood samples for glucose and hemoglobin A1c analyses were collected after an overnight fast of at least 6 hours.

| **Fasting blood glucose (FBG) (mg/dl), hemoglobin A1c (HbA1c) (%)** | **Score** |
| --- | --- |
| No history of diabetes and FBG <100.0 (or HbA1c <5.70) | 100 |
| No diabetes and FBG 100.0-125.9 (or HbA1c 5.70-6.49) (prediabetes) | 60 |
| Diabetes with HbA1c <7.0 | 40 |
| Diabetes with HbA1c 7.0-7.9 | 30 |
| Diabetes with HbA1c 8.0-8.9 | 20 |
| Diabetes with Hb A1c 9.0-9.9 | 10 |
| Diabetes with HbA1c ≥10.0 | 0 |
| Diabetes was considered as: 1) taking antidiabetic medication, or HbA1c ≥6.50%, or FBG ≥126.0 mg/dl. | |

**Life’s Essential 8 Factor score**

The calculation of the Life’s Essential 8 Factor (LE8 Factor) score is consistent with that published by the American Heart Association.^8^ All 4 components within the LE8 Factor score were scored from 0 (worst cardiovascular health) to 100 (best cardiovascular health). In line with the American Heart Association recommendations, the LE8 Factor score was then calculated as the unweighted average of the four components, yielding an overall score ranging from 0 to 100.

- LE8 Factor score=sum of all 4 LE8 factor components / 4.

Additionally, a normalized LE8 Factor score was calculated by summing the Z-scores of the four components, which was then standardized into a single Z-score.

**References**
1. Lloyd-Jones DM, Allen NB, Anderson CAM, Black T, Brewer LC, Foraker RE, et al. Life’s Essential 8: Updating and Enhancing the American Heart Association’s Construct of Cardiovascular Health: A Presidential Advisory From the American Heart Association. Circulation. 2022. doi:10.1161/CIR.0000000000001078
