# Supplementary figures and images for: Validity of the International Fitness Scale (IFIS) and its associations with cardiometabolic health and body composition in adults with type 2 diabetes: A cross-sectional study
Source: PLoS One. 2026 Jan 6;21(1):e0339364. doi: 10.1371/journal.pone.0339364 (PMC12774367; doi:10.1371/journal.pone.0339364)

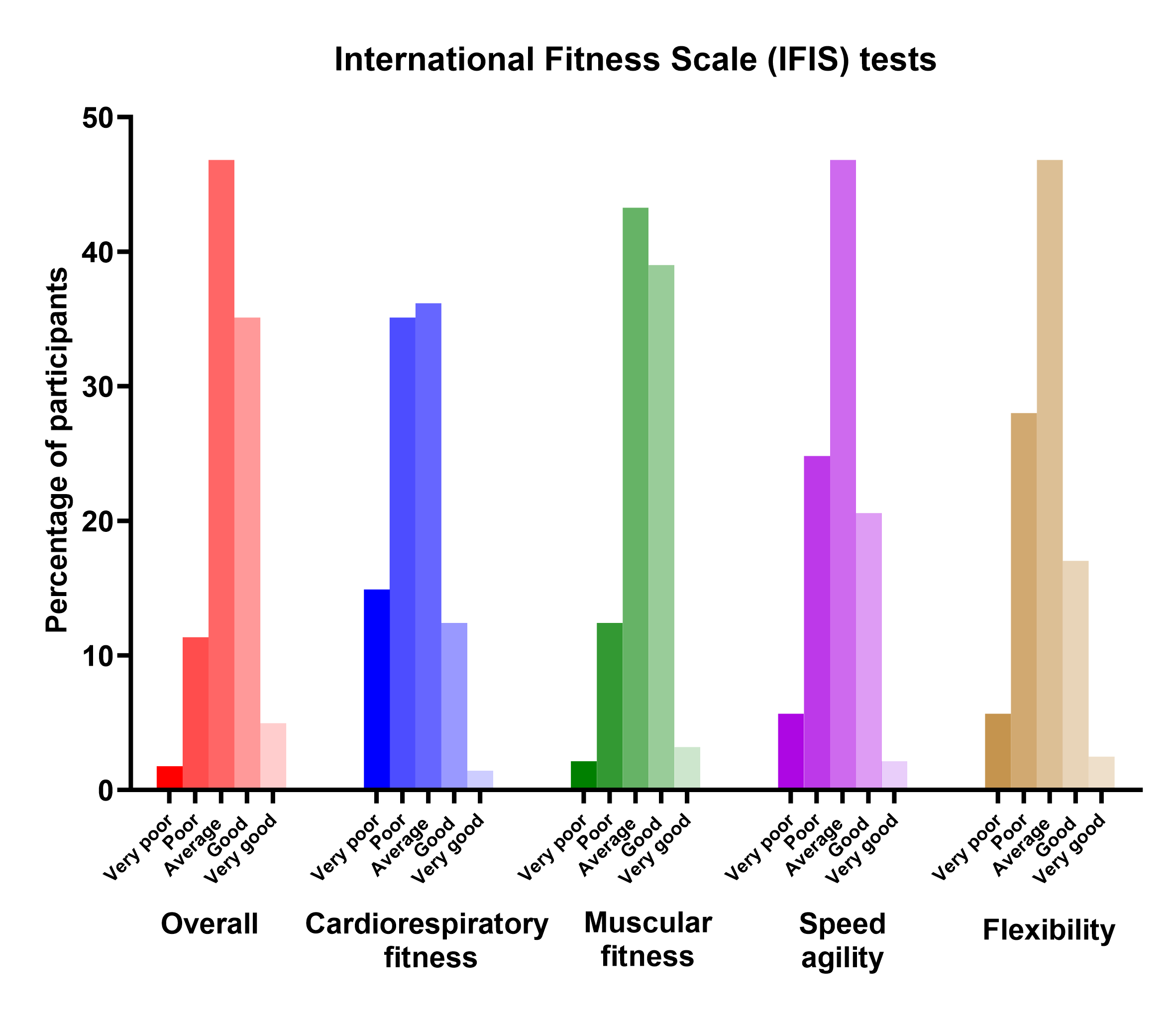

Supplement: S1 Fig — IFIS: International Fitness Scale. (TIF) [file pone.0339364.s002.tif]

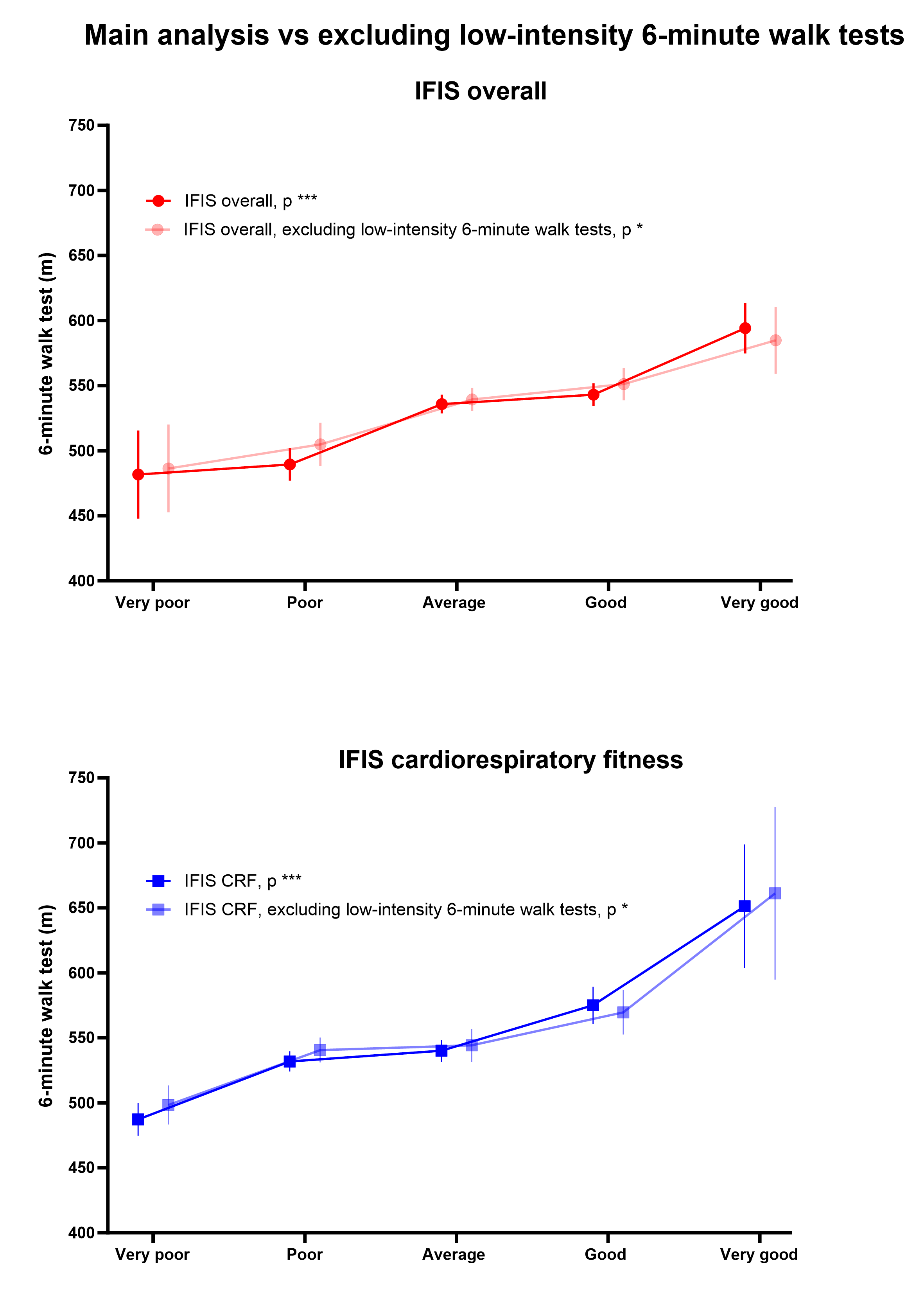

Supplement: S2 Fig — The graphic depicts means with standard errors for the 6-minute walk test according to different levels of the IFIS scores. Analyses of covariance (ANCOVA) are adjusted for sex and age. Presumably low-intensity 6-minute walk tests are considered those achieving less than 60% of the predicted maximal heart rate after test calculated with the Tanaka’s formula. Significance level for each IFIS test is indicated as follows: ***, p < 0.001; **, p < 0.01; *, p < 0.05; and ns, non-significant. To allow comparability, population is restricted to those having information on the heart rate after the 6-minute walk test, IFIS overall and IFIS CRF (n = 193), IFIS overall and IFIS CRF excluding low-intensity tests (n = 114). CRF: cardiorespiratory fitness, IFIS: International Fitness Scale. (TIF) [file pone.0339364.s003.tif]

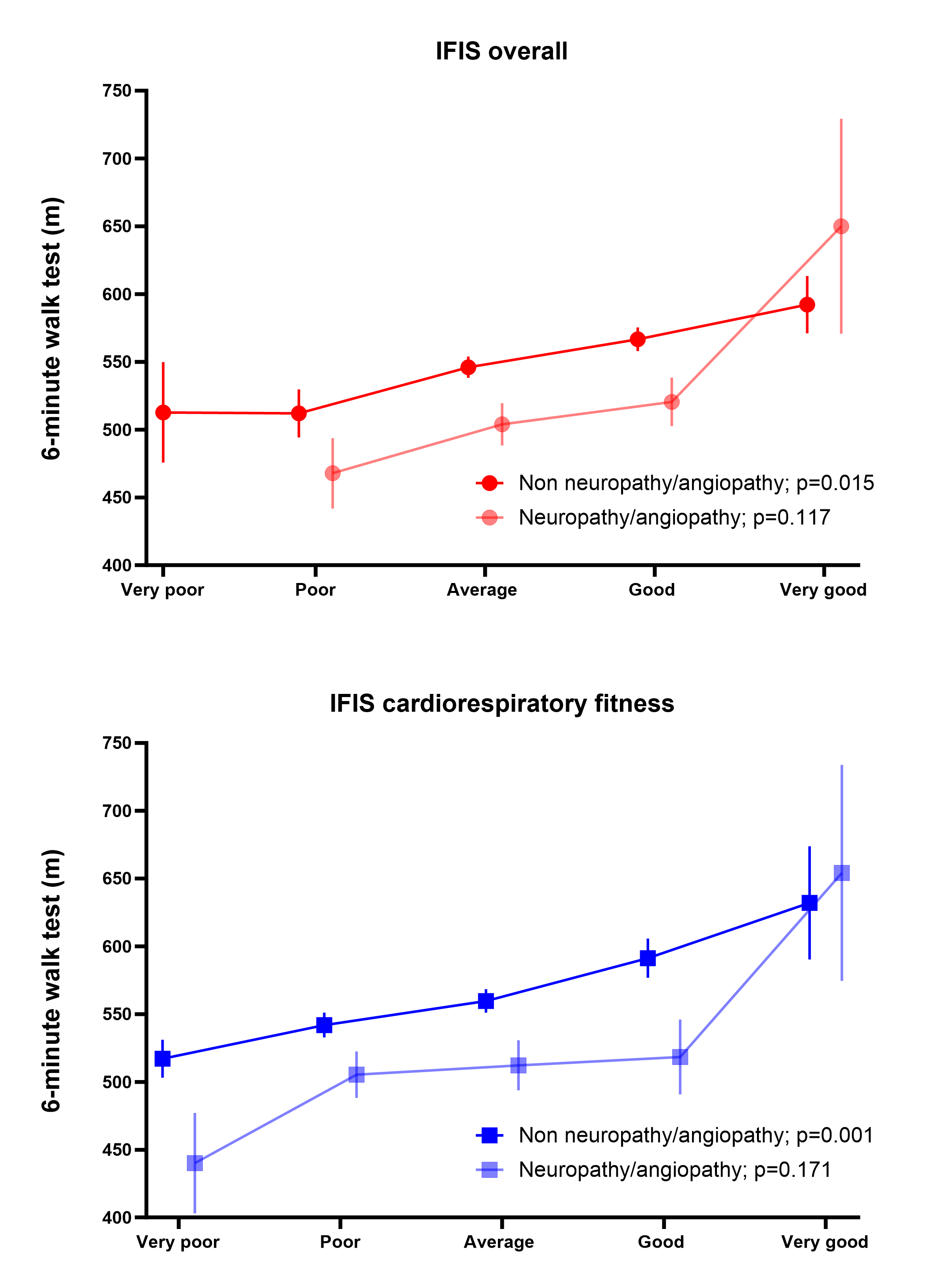

Supplement: S3 Fig — The graphic depicts means with standard errors for the 6-minute walk test according to different levels of the IFIS scores. Analyses of covariance (ANCOVA) are adjusted for sex and age. No neuropathy/angiopathy, n = 193. Neuropathy/angiopathy, n = 54. IFIS: International Fitness Scale. (TIF) [file pone.0339364.s004.tif]

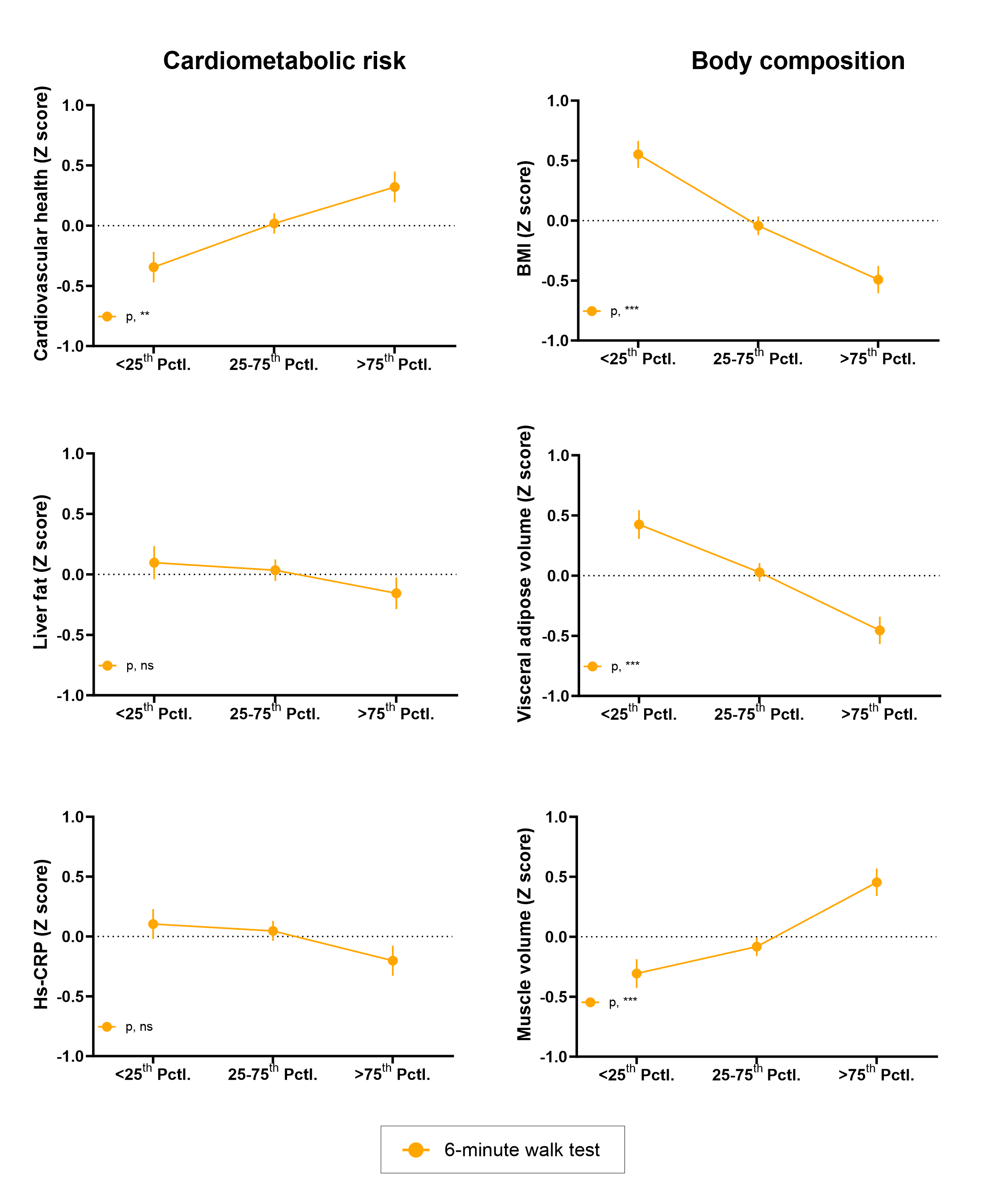

Supplement: S4 Fig — The graphic depicts means and standard errors for the analyses of covariance (ANCOVA) adjusted for sex and age. The variables liver fat and hs-CRP were natural-logarithmically transformed. All variables are presented as Z-scores (mean = 0, standard deviation = 1). The 6-minute walk tests are categorized as <25th percentile, 25–75th percentile, and >75th percentile. Significance level for each 6-minute walk test is indicated as follows: ***, p < 0.001; **, p < 0.01; *, p < 0.05; and ns, non-significant. BMI: body mass index, hs-CRP: high-sensitive C-reactive protein, Pctl: percentile. (TIF) [file pone.0339364.s005.tif]

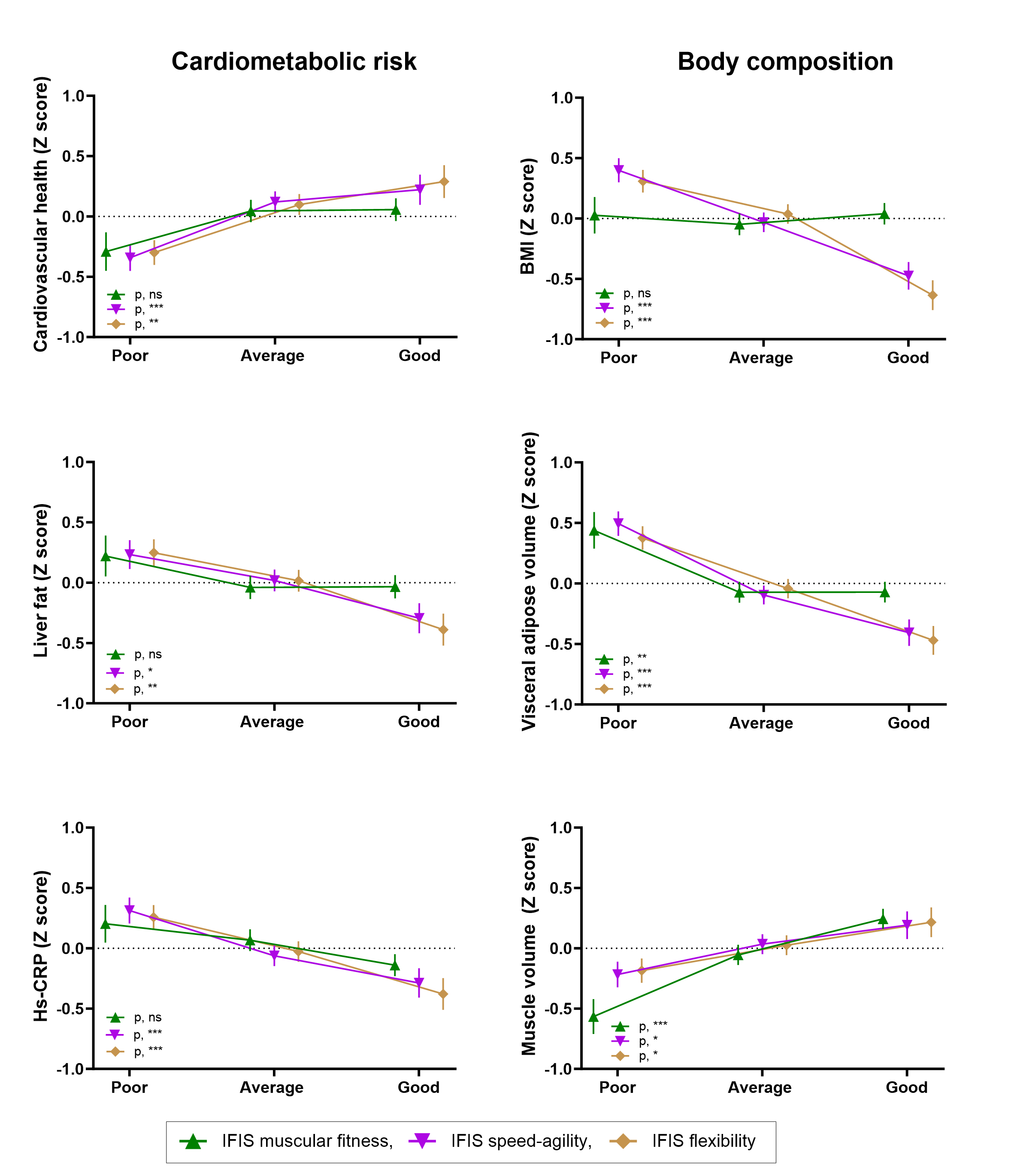

Supplement: S5 Fig — The graphic depicts means and standard errors for the analyses of covariance (ANCOVA) adjusted for sex and age. The variables liver fat and hs-CRP were natural-logarithmically transformed. All variables are presented as Z-scores (mean = 0, standard deviation = 1). IFIS scores are categorized as poor (very poor/poor), average and good (good/very good). Significance level for each IFIS test is indicated as follows: ***, p < 0.001; **, p < 0.01; *, p < 0.05; and ns, non-significant. BMI: body mass index, hs-CRP: high-sensitivity C-reactive protein, IFIS: International Fitness Scale. (TIF) [file pone.0339364.s006.tif]

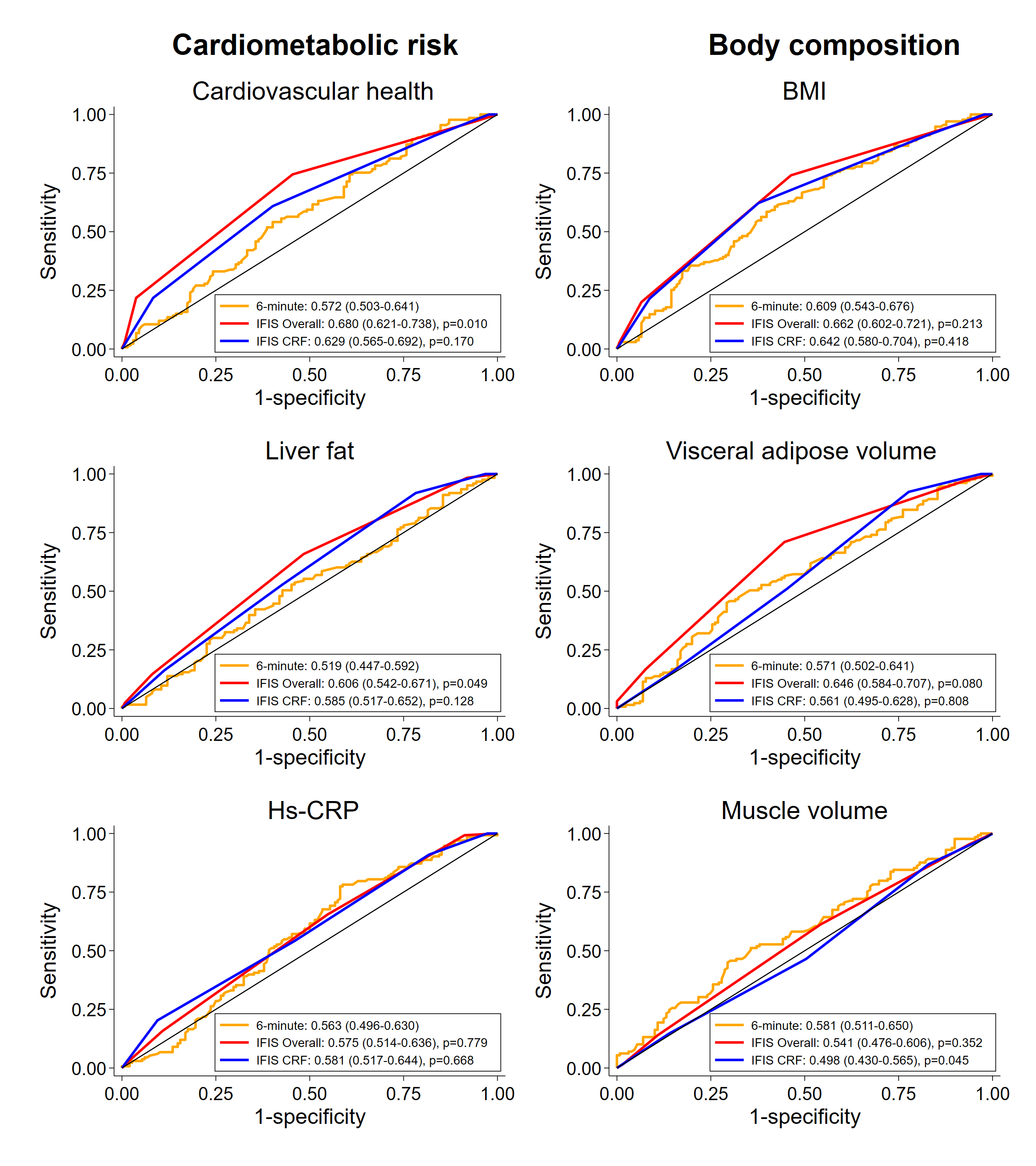

Supplement: S6 Fig — The legends indicate the AUCs with corresponding 95% confidence intervals. The p-values represent comparisons of the AUCs between each specific IFIS test (IFIS overall and IFIS cardiorespiratory fitness) and the 6-minute walk test, performed using the DeLong test. Outcomes are dichotomized at the median of the Z-scores (0 indicating worse health, 1 indicating better health). AUC: area under curve, BMI: body mass index, CRF: cardiorespiratory fitness, hs-CRP: high-sensitive C-reactive protein, IFIS: International Fitness Scale, ROC: receiver operating characteristic curve. (TIF) [file pone.0339364.s007.tif]

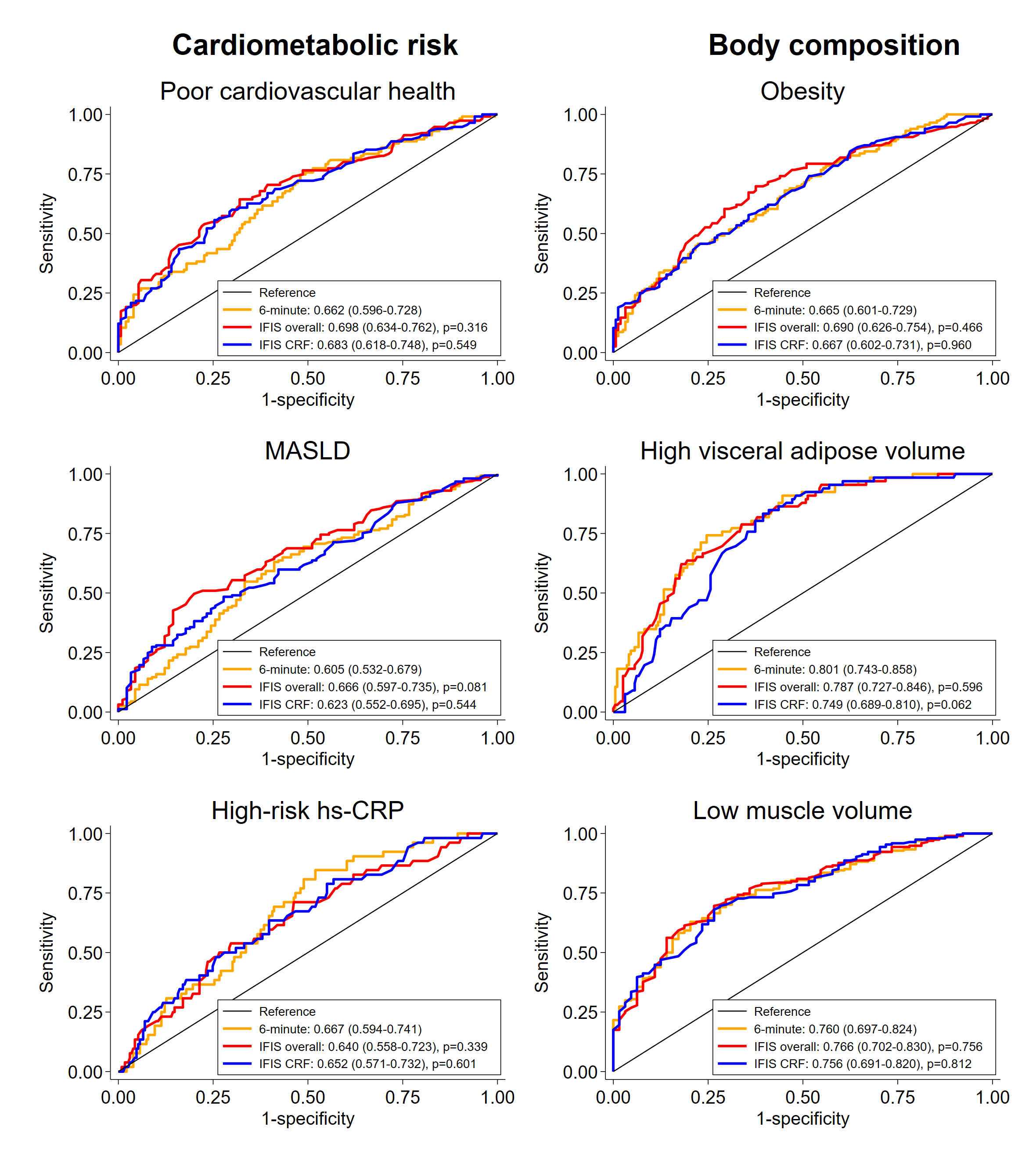

Supplement: S7 Fig — The legends indicate AUCs with corresponding 95% confidence intervals. The p-values represent comparisons of the AUCs between the probabilities derived from the adjusted logistic models of the IFIS scores (IFIS overall and IFIS cardiorespiratory fitness) and the 6-minute walk test, performed using the DeLong test. All models are adjusted for age (continuous) and sex (female/male). Outcomes were dichotomized using established thresholds: poor cardiovascular health (<50 points), MASLD (≥5% liver fat), high-risk hs-CRP (>3.0 mg/L), obesity (BMI ≥ 30 kg/m²), high visceral adipose tissue (≥75th percentile), and low muscle volume (<25th percentile). AUC: area under curve, CRF: cardiorespiratory fitness, hs-CRP: high-sensitivity C-reactive protein, IFIS: International Fitness Scale, MASLD: metabolic dysfunction-associated steatotic liver disease, ROC: receiver operating characteristic curve. (TIF) [file pone.0339364.s008.tif]
