# Supplementary material for: Validity of the International Fitness Scale (IFIS) and its associations with cardiometabolic health and body composition in adults with type 2 diabetes: A cross-sectional study
Source: PLoS One. 2026 Jan 6;21(1):e0339364. doi: 10.1371/journal.pone.0339364 (PMC12774367; doi:10.1371/journal.pone.0339364)
Supplement: S1 Table — Categorical variables are depicted as frequency (percentages). Continuous variables normally and not normally distributed (marked with a) are depicted as mean ± standard deviation and median ± interquartile range, respectively. Incretin-based medications refer to the use of glucagon-like peptide-1 receptor agonists (GLP-1 RAs) and/or inhibitors of the enzyme dipeptidyl peptidase-4 (DPP-4 inhibitors). Thigh fat-free muscle volume (Z-score) is standardized by sex, thigh length, height, and BMI from the general population. BMI: body mass index, hs-CRP: high-sensitivity C-reactive protein, IFIS: International Fitness Scale, SGLT2: sodium-glucose co-transporter 2 medication, T2DM: type 2 diabetes mellitus. (DOCX) [file pone.0339364.s009.docx]

| **S1 Table. Clinical characteristics of the participants by IFIS overall fitness scores, 5 categories.** | | | | | |
| --- | --- | --- | --- | --- | --- |
|  | **IFIS overall fitness (n=282)** | | | | |
|  | **Very poor, n=5** | **Poor, n=32** | **Average, n=132** | **Good, n=99** | **Very good, n=14** |
| **Sex (female, %)** | 4 (80) | 14 (43.8) | 51 (38.6) | 33 (33.3) | 5 (35.7) |
| **Age (years)** | 58.0 ± 7.9 | 60.5 ± 10.3 | 63.4 ± 8.0 | 65.3 ± 6.9 | 64.2 ± 9.1 |
| **Duration of T2DM (years)** | 6.4 ± 4.0 | 7.4 ± 4.7 | 8.4 ± 7.6 | 9.2 ± 6.7 | 6.4 ± 7.1 |
| **Current use of diabetes medications** |  |  |  |  |  |
| Insulin | 1 (20.0) | 4 (13.3) | 32 (25.2) | 16 (16.7) | 2 (15.4) |
| Metformin | 3 (60.0) | 24 (77.4) | 99 (75.6) | 80 (80.8) | 11 (78.6) |
| Sulfonylurea | 1 (20.0) | 5 (16.1) | 12 (9.3) | 7 (7.3) | - |
| SGLT2-inhibitor | 2 (40.0) | 10 (31.3) | 42 (33.3) | 24 (25) | 2 (15.4) |
| Incretin-based | 1 (20.0) | 3 (9.7) | 9 (7.0) | 10 (10.4) | - |
| **Smoking** |  |  |  |  |  |
| Never smoked | - | 14 (43.8) | 59 (44.7) | 57 (57.6) | 8 (57.1) |
| Current smoker | - | 2 (6.3) | 4 (3.0) | 4 (4.0) | 1 (7.1) |
| Ex-smoker | 5 (100.0) | 16 (50.0) | 69 (52.3) | 38 (38.4) | 5 (35.7) |
| **Physical activity** |  |  |  |  |  |
| Moderate-vigorous PA (time spent in 1-5 min bouts, min/day) | 7.5 ± 8.8 | 7.8 ± 9.4 | 11.4 ± 12.1 | 11.2 ± 8.3 | 19.3 ± 15.4 |
| Light PA (time spent in 10 min bouts, min/day) | 1.8 ± 2.2 | 3.9 ± 9.0 | 2.8 ± 4.1 | 4.9 ± 7.1 | 8.3 ± 11.1 |
| Sedentary time (time spent in 30 min bouts, min/day) | 579.5 ± 162.3 | 601.9 ± 203.0 | 556.8 ± 162.4 | 540.7 ± 161.4 | 502.6 ± 170.5 |
| **Diabetic foot risk** |  |  |  |  |  |
| Healthy | 4 (80.0) | 18 (64.3) | 87 (71.9) | 72 (73.5) | 12 (85.7) |
| Neuropahy/angiopathy | - | 9 (32.1) | 25 (20.7) | 19 (19.4) | 1 (7.1) |
| Previous foot ulcers | 1 (20.0) | 1 (3.6) | 9 (7.4) | 7 (7.1) | 1 (7.1) |
| **6-minute walk test (meters)** | 508.2 ± 95.3 | 500.0 ± 77.9 | 533.1 ± 87.8 | 552.3 ± 84.0 | 598.9 ± 51.5 |
| **6-minute walk test** |  |  |  |  |  |
| <25^th^ percentile; <489 meters | 2 (40.0) | 12 (37.5) | 36 (27.3) | 21 (21.2) | - |
| 25-75^th^ percentile; 489-595 meters | 2 (40.0) | 16 (50.0) | 66 (50.0) | 53 (53.5) | 5 (35.7) |
| >75^th^ percentile; ≥596 meters | 1 (20.0) | 4 (12.5) | 30 (22.7) | 25 25.3) | 9 (64.3) |
| **IFIS overall fitness** | 1.0 ± 0.0 | 2.0 ± 0.0 | 3.0 ± 0.0 | 4.0 ± 0.0 | 5.0 ± 0.0 |
| **IFIS cardiorespiratory fitness** | 1.0 ± 0.0 | 1.5 ± 0.5 | 2.2 ± 0.7 | 3.0 ± 0.8 | 4.0 ± 0.8 |
| **IFIS muscular fitness** | 1.4 ± 0.5 | 2.6 ± 0.8 | 3.2 ± 0.7 | 3.6 ± 0.6 | 4.3 ± 0.6 |
| **IFIS speed-agility** | 1.0 ± 0.0 | 2.1 ± 0.7 | 2.7 ± 0.8 | 3.3 ± 0.7 | 3.7 ± 0.6 |
| **IFIS flexibility** | 1.0 ± 0.0 | 2.1 ± 0.7 | 2.7 ± 0.7 | 3.3 ± 0.8 | 3.4 ± 0.8 |
| **Cardiovascular health score** | 39.8 ± 12.4 | 44.5 ± 9.2 | 50.3 ± 13.3 | 56.2 ± 11.3 | 56.9 ± 13.6 |
| **Liver fat (%)^a^** | 18.5 ± 9.8 | 14.9 ± 10.9 | 9.2 ± 11.7 | 9.1 ± 8.5 | 5.9 ± 6.2 |
| **Hs-CRP (mg/L)^a^** | 2.7 ± 3.6 | 2.0 ± 1.9 | 1.3 ± 1.8 | 2.1 ± 3.6 | 0.6 ± 0.6 |
| **BMI (kg/m2)** | 30.9 ± 2.2 | 33.0 ± 4.7 | 30.2 ± 4.5 | 27.4 ± 3.5 | 27.6 ± 4.4 |
| **Visceral adipose tissue volume (L)** | 8.3 ± 2.5 | 7.5 ± 2.7 | 6.5 ± 2.5 | 5.3 ± 2.5 | 4.8 ± 2.8 |
| **Thigh fat-free muscle volume (Z-score)** | 0.2 ± 0.7 | -0.1 ± 1.2 | -0.10 ± 1.01 | 0.1 ± 1.0 | 0.7 ± 1.3 |
| Categorical variables are depicted as frequency (percentages). Continuous variables normally and not normally distributed (marked with ^a^) are depicted as mean ± standard deviation and median ± interquartile range, respectively.  Incretin-based medications refer to the use of glucagon-like peptide-1 receptor agonists (GLP-1 RAs) and/or inhibitors of the enzyme dipeptidyl peptidase-4 (DPP-4 inhibitors).  Tigh fat-free muscle volume (Z-score) is standardized by sex, length, height, and BMI from the general population.  BMI: body mass index, hs-CRP: high-sensitivity C-reactive protein, IFIS: International Fitness Scale, PA: physical activity, min: minute, SGLT2: sodium-glucose co-transporter 2 medication, T2DM: type 2 diabetes mellitus. | | | | | |
