# Supplementary material for: Validity of the International Fitness Scale (IFIS) and its associations with cardiometabolic health and body composition in adults with type 2 diabetes: A cross-sectional study
Source: PLoS One. 2026 Jan 6;21(1):e0339364. doi: 10.1371/journal.pone.0339364 (PMC12774367; doi:10.1371/journal.pone.0339364)
Supplement: S2 Table — Data are presented as means and 95% confidence intervals. Adjusted models are adjusted by age and sex. Superscripts indicate statistically significant Tukey’s pairwise comparisons (p < 0.05) for the means of the 6-minute walk test across categories of the IFIS scores: VP (Very poor), P (Poor), A (Average), G (Good), and VG (Very good). For example, in IFIS overall fitness, for the unadjusted model, those rating their overall fitness as “Poor” had significant differences in the 6-minute walk test compared to those rating their overall fitness as “Good” or “Very good”. CI: confidence interval, IFIS: International Fitness Scale. (DOCX) [file pone.0339364.s010.docx]

| **S2 Table . Differences in the 6-minute walk test according to categories of self-reported (IFIS) physical fitness scores.** | | | | | | |
| --- | --- | --- | --- | --- | --- | --- |
|  | **6-minute walk test** | | | | | |
|  | **Unadjusted** | | | **Adjusted** | | |
|  | **Category** | **Mean** | **95% CI** | **Category** | **Mean** | **95% CI** |
| **IFIS**  **overall fitness** | Very poor ^-^ | 508.2 | (434.1, 582.3) | Very poor ^-^ | 506.5 | (440.8, 572.2) |
|  | Poor ^G, VG^ | 500.0 | (470.7, 529.3) | Poor ^A, G, VG^ | 489.8 | (463.9, 515.8) |
|  | Average ^VG^ | 533.1 | (518.7, 547.6) | Average ^P, VG^ | 532.3 | (519.7, 545.0) |
|  | Good ^P^ | 552.3 | (535.6, 568.9) | Good ^P^ | 556.6 | (541.8, 571.3) |
|  | Very good ^P, A^ | 598.9 | (554.6, 643.1) | Very good ^P, A^ | 600.1 | (561.2, 638.9) |
| **IFIS cardiorespiratory fitness** | Very poor ^A, G, VG^ | 494.9 | (469.7, 520.1) | Very poor ^P,^ ^A, G, VG^ | 492.0 | (468.7, 515.4) |
|  | Poor ^G^ | 531.5 | (515.1, 547.9) | Poor ^VP, G, VG^ | 533.8 | (519.2, 548.4) |
|  | Average ^VP^ | 546.9 | (530.7, 563.1) | Average ^VP^ | 547.1 | (532.5, 561.7) |
|  | Good ^VP, P^ | 579.6 | (552.0, 607.2) | Good ^VP^ | 574.0 | (549.4, 598.7) |
|  | Very good ^VP^ | 624.5 | (542.9, 706.1) | Very good ^VP, P^ | 641.5 | (568.9, 714.1) |
| Data are presented as means and 95% confidence intervals. Adjusted models are adjusted by age and sex. Superscripts indicate statistically significant Tukey’s pairwise comparisons (p<0.05) for the means of the 6-minute walk test across categories of the IFIS scores: VP (Very poor), P (Poor), A (Average), G (Good), and VG (Very good). For example, in IFIS overall fitness, for the unadjusted model, those rating their overall fitness as “Poor” had significant differences in the 6-minute walk test compared to those rating their overall fitness as “Good” or “Very good”.  CI: confidence interval, IFIS: International Fitness Scale. | | | | | | |
