# Supplementary material for: Validity of the International Fitness Scale (IFIS) and its associations with cardiometabolic health and body composition in adults with type 2 diabetes: A cross-sectional study
Source: PLoS One. 2026 Jan 6;21(1):e0339364. doi: 10.1371/journal.pone.0339364 (PMC12774367; doi:10.1371/journal.pone.0339364)
Supplement: S3 Table — Population-predicted mean 6-MWT distance represents the weighted average estimated for the entire study population, calculated as 522.9 meters according to the equations by Enright & Sherrill, based on population-level age, weight, and height in women and men. Individualized predicted average 6-MWT distance is calculated for each participant using the Enright & Sherrill equations, based on their age, sex, weight, and height. 6-MWT: 6-minute walk test (meters), AUC: area under receiver operating characteristic curve, CI: confidence interval, DOR: diagnostic odds ratio, IFIS: International Fitness Scale, LR: likelihood ratio, NPV: negative predictive value, PPV: positive predictive value. (DOCX) [file pone.0339364.s011.docx]

| **S3 Table. Diagnostic performance of the IFIS cardiorespiratory fitness test in identifying different levels of functional capacity as defined by the 6-minute walk test.** | | | | | | | | | | | |
| --- | --- | --- | --- | --- | --- | --- | --- | --- | --- | --- | --- |
|  | **<Population predicted mean 6-MWT (522.9 meters)** | | | | | | | | | | |
|  | **IFIS cardiorespiratory fitness** | | | | | | | | | | |
|  | **Very poor** | | | **Very poor, poor** | | **Very poor, poor, average** | | | **Very poor, poor, average, good** | | |
| **Prevalence** | 40.4 | (34.6, 46.4) | | 40.4 | (34.6, 46.4) | 40.4 | (34.6, 46.4) | | 40.4 | (34.6, 46.4) | |
| **Sensitivity (%)** | 21.9 | (14.7, 30.6) | | 58.8 | (49.2, 67.9) | 93.9 | (87.8, 97.5) | | 100.0 | (96.8, 100.0) | |
| **Specificity (%)** | 89.9 | (84.3, 94) | | 56.0 | (48.1, 63.6) | 19.0 | (13.4, 25.8) | | 2.4 | (0.7, 6.0) | |
| **LR+** | 2.17 | (1.23, 3.83) | | 1.33 | (1.06, 1.68) | 1.16 | (1.06, 1.26) | | 1.02 | (1.00, 1.05) | |
| **LR-** | 0.87 | (0.78, 0.97) | | 0.74 | (0.57, 0.95) | 0.32 | (0.15, 0.70) | | - | - | |
| **DOR** | 2.5 | (1.29, 4.84) | | 1.81 | (1.12, 2.93) | 3.6 | (1.56, 8.28) | | - | (0.71, - ) | |
| **PPV (%)** | 59.5 | (43.3, 74.4) | | 47.5 | (39.1, 56.1) | 44.0 | (37.7, 50.5) | | 41.0 | (35.2, 47.0) | |
| **NPV (%)** | 62.9 | (56.5, 69) | | 66.7 | (58.2, 74.4) | 82.1 | (66.5, 92.5) | | 100.0 | (39.8, 100.0) | |
|  | **Impaired functional capacity, <80% individualized predicted 6-MWT** | | | | | | | | | | |
|  | **IFIS cardiorespiratory fitness** | | | | | | | | | | |
|  | **Very poor** | | | **Very poor, poor** | | **Very poor, poor, average** | | | **Very poor, poor, average, good** | | |
| **Prevalence** | 5.9 | (3.4, 9.4) | | 5.9 | (3.4, 9.4) | 5.9 | (3.4, 9.4) | | 5.9 | (3.4, 9.4) | |
| **Sensitivity (%)** | 25.0 | (7.3, 52.4) | | 56.2 | (29.9, 80.2) | 87.5 | (61.7, 98.4) | | 100.0 | (79.4, 100.0) | |
| **Specificity (%)** | 85.9 | (81.0, 89.9) | | 51.0 | (44.7, 57.3) | 13.3 | (9.4, 18.1) | | 1.2 | (0.2, 3.4) | |
| **LR+** | 1.77 | (0.72, 4.36) | | 1.15 | (0.73, 1.80) | 1.01 | (0.83, 1.22) | | 1.01 | (1.00, 1.03) | |
| **LR-** | 0.87 | (0.66, 1.16) | | 0.86 | (0.49, 1.52) | 0.94 | (0.25, 3.56) | | - | - | |
| **DOR** | 2.03 | (0.65, 6.33) | | 1.34 | (0.50, 3.57) | 1.08 | (0.26, - ) | | - | (0.05, - ) | |
| **PPV (%)** | 10.0 | (2.8, 23.7) | | 6.7 | (3.1, 12.4) | 6.0 | (3.3, 9.8) | | 6.0 | (3.5, 9.5) | |
| **NPV (%)** | 94.8 | (91.1, 97.3) | | 94.9 | (89.8, 97.9) | 94.4 | (81.3, 99.3) | | 100.0 | (29.2, 100) | |
|  | | | **<Population predicted**  **mean 6-MWT (522.9 meters)** | | | | | **<80% individualized predicted 6-MWT** | | |  |
| **AUC IFIS cardiorespiratory fitness** | | | 0.615 (0.552, 0.678) | | | | | 0.556 (0.404, 0.708) | | |  |
| **AUC IFIS muscular fitness** | | | 0.573 (0.510, 0.636) | | | | | 0.708 (0.592, 0.823) | | |  |
| **AUC IFIS speed-agility** | | | 0.611 (0.550, 0.672) | | | | | 0.614 (0.460, 0.767) | | |  |
| **AUC IFIS flexibility** | | | 0.596 (0.535, 0.658) | | | | | 0.597 (0.429, 0.765) | | |  |
| **AUC IFIS overall fitness** | | | 0.614 (0.553, 0.675) | | | | | 0.613 (0.483, 0.744) | | |  |
| Population-predicted mean 6-MWT distance represents the weighted average estimated for the entire study population, calculated as 522.9 meters according to the equations by Enright & Sherrill, based on population-level age, weight, and height in women and men.  Individualized predicted average 6-MWT distance is calculated for each participant using the Enright & Sherrill equations, based on their age, sex, weight, and height.  6-MWT: 6-minute walk test (meters), AUC: area under receiver operating characteristic curve, CI: confidence interval, DOR: diagnostic odds ratio, IFIS: International Fitness Scale, LR: likelihood ratio, NPV: negative predictive value, PPV: positive predictive value. | | | | | | | | | | | |
