# Supplementary material for: Validity of the International Fitness Scale (IFIS) and its associations with cardiometabolic health and body composition in adults with type 2 diabetes: A cross-sectional study
Source: PLoS One. 2026 Jan 6;21(1):e0339364. doi: 10.1371/journal.pone.0339364 (PMC12774367; doi:10.1371/journal.pone.0339364)
Supplement: S4 Table — Data are presented as means and 95% confidence intervals. Adjusted models are adjusted by age and sex. Superscripts indicate statistically significant Tukey’s pairwise comparisons (p < 0.05) for the means of the 6-minute walk test across categories of the IFIS scores: VP (Very poor), P (Poor), A (Average), G (Good), and VG (Very good). For example, in IFIS overall fitness, for the unadjusted model, those rating their overall fitness as “Poor” had significant differences in the 6-minute walk test compared to those rating their overall fitness as “Good” or “Very good”. CI: confidence interval, IFIS: International Fitness Scale. (DOCX) [file pone.0339364.s012.docx]

| **S4 Table. Differences in the 6-minute walk test according to categories of self-reported (IFIS) physical fitness scores comparing diabetic participants with and without neuropathy/angiopathy.** | | | | | | |
| --- | --- | --- | --- | --- | --- | --- |
|  | **6-minute walk test** | | | | | |
|  | **Non neuropathy/angiopathy** | | | **Neuropathy/angiopathy** | | |
|  | **Category** | **Mean** | **95% CI** | **Category** | **Mean** | **95% CI** |
| **IFIS**  **overall fitness** | Very poor | 512.7 | (439.5, 585.9) | Very poor | - | - |
|  | Poor | 512.0 | (477.1, 546.8) | Poor | 467.8 | (415.6, 520.1) |
|  | Average | 546.0 | (530.6, 561.5) | Average | 503.9 | (472.6, 535.2) |
|  | Good | 566.6 | (549.4, 583.9) | Good | 520.5 | (484.5, 556.4) |
|  | Very good | 592.3 | (550.6, 633.9) | Very good | 650.1 | (490.8, 809.4) |
| **IFIS cardiorespiratory fitness** | Very poor | 517.1 | (489.5, 544.8) | Very poor | 440.1 | (365.6, 514.6) |
|  | Poor | 542.0 | (524.0, 559.9) | Poor | 505.3 | (470.8, 539.9) |
|  | Average | 559.7 | (542.7, 576.8) | Average | 512.2 | (475.0, 549.4) |
|  | Good | 591.2 | (562.7, 619.8) | Good | 518.4 | (462.8, 574.0) |
|  | Very good | 632.1 | (549.8, 714.4) | Very good | 654.2 | (494.1, 814.3) |
| Data are presented as means and 95% confidence intervals. Adjusted models are adjusted by age and sex. Superscripts indicate statistically significant Tukey’s pairwise comparisons (p<0.05) for the means of the 6-minute walk test across categories of the IFIS scores: VP (Very poor), P (Poor), A (Average), G (Good), and VG (Very good). For example, in IFIS overall fitness, for the unadjusted model, those rating their overall fitness as “Poor” had significant differences in the 6-minute walk test compared to those rating their overall fitness as “Good” or “Very good”.  CI: confidence interval, IFIS: International Fitness Scale. | | | | | | |
|  | | | | | | |
