# Supplementary material for: Validity of the International Fitness Scale (IFIS) and its associations with cardiometabolic health and body composition in adults with type 2 diabetes: A cross-sectional study
Source: PLoS One. 2026 Jan 6;21(1):e0339364. doi: 10.1371/journal.pone.0339364 (PMC12774367; doi:10.1371/journal.pone.0339364)
Supplement: S5 Table — Data are presented as mean and 95% confidence intervals. Adjusted models are adjusted by age and sex. Superscripts indicate statistically significant Tukey’s pairwise comparisons (p < 0.05) for the Z-scores of cardiometabolic outcomes across categories of the IFIS scores: P (Poor), A (Average), and G (Good). For example, in IFIS overall fitness, for the unadjusted model, those rating their overall fitness as “Poor” had significant differences in the Z-score for cardiovascular health compared to those rating their overall fitness as “Good”. CI: confidence interval, hs-CRP: high-sensitivity C-reactive protein, IFIS: International Fitness Scale. (DOCX) [file pone.0339364.s013.docx]

| **S5 Table .** **Differences in cardiometabolic outcomes (Z-scores) according to categories of self-reported (IFIS) physical fitness scores.** | | | | | | |
| --- | --- | --- | --- | --- | --- | --- |
|  | **Unadjusted** | | | | **Adjusted** | |
|  | **Category** | **Mean** | **95% CI** | **Category** | **Mean** | **95% CI** |
| **Cardiovascular health (Z-score)** | | | | | | |
| **IFIS**  **overall fitness** | Poor ^-,G^ | -0.538 | (-0.863, -0.214) | Poor ^-,G^ | -0.515 | (-0.844, -0.186) |
|  | Average ^-,G^ | -0.120 | (-0.289, 0.050) | Average ^-,G^ | -0.115 | (-0.284, 0.054) |
|  | Good ^P,A^ | 0.314 | (0.130, 0.497) | Good ^P,A^ | 0.301 | (0.115, 0.487) |
| **IFIS cardiorespiratory fitness** | Poor ^A,-^ | -0.190 | (-0.357, -0.022) | Poor ^A,-^ | -0.189 | (-0.359, -0.020) |
|  | Average ^P,-^ | 0.208 | (0.011, 0.405) | Average ^P,-^ | 0.208 | (0.010, 0.406) |
|  | Good ^-,-^ | 0.154 | (-0.178, 0.487) | Good ^-,-^ | 0.152 | (-0.181, 0.485) |
| **IFIS**  **muscular fitness** | Poor ^-,-^ | -0.289 | (-0.604, 0.025) | Poor ^-,-^ | -0.291 | (-0.606, 0.023) |
|  | Average ^-,-^ | 0.048 | (-0.135, 0.231) | Average ^-,-^ | 0.045 | (-0.137, 0.227) |
|  | Good ^-,-^ | 0.052 | (-0.135, 0.238) | Good ^-,-^ | 0.056 | (-0.129, 0.241) |
| **IFIS**  **speed-agility** | Poor ^A,G^ | -0.374 | (-0.585, -0.162) | Poor ^A,G^ | -0.342 | (-0.557, -0.128) |
|  | Average ^P,-^ | 0.137 | (-0.035, 0.309) | Average ^P,-^ | 0.120 | (-0.053, 0.294) |
|  | Good ^P,-^ | 0.230 | (-0.017, 0.476) | Good ^P,-^ | 0.222 | (-0.025, 0.469) |
| **IFIS**  **flexibility** | Poor ^A,G^ | -0.321 | (-0.523, -0.118) | Poor ^A,G^ | -0.299 | (-0.502, -0.096) |
|  | Average ^P,-^ | 0.111 | (-0.062, 0.283) | Average ^P,-^ | 0.098 | (-0.074, 0.271) |
|  | Good ^P,-^ | 0.297 | (0.028, 0.566) | Good ^P,-^ | 0.288 | (0.020, 0.557) |
| **Liver fat (Z-score)** | | | | | | |
| **IFIS**  **overall fitness** | Poor ^-,G^ | 0.458 | (0.099, 0.817) | Poor ^-,G^ | 0.417 | (0.056, 0.777) |
|  | Average ^-,G^ | 0.170 | (-0.009, 0.349) | Average ^-,G^ | 0.161 | (-0.018, 0.339) |
|  | Good ^P,A^ | -0.302 | (-0.487, -0.118) | Good ^P,A^ | -0.281 | (-0.467, -0.096) |
| **IFIS cardiorespiratory fitness** | Poor ^-,G^ | 0.177 | (0.000, 0.354) | Poor ^-,G^ | 0.153 | (-0.027, 0.333) |
|  | Average ^-,G^ | -0.027 | (-0.228, 0.173) | Average ^-,G^ | 0.003 | (-0.200, 0.207) |
|  | Good ^P,A^ | -0.497 | (-0.814, -0.181) | Good ^P,A^ | -0.495 | (-0.810, -0.180) |
| **IFIS**  **muscular fitness** | Poor ^-,-^ | 0.249 | (-0.084, 0.582) | Poor ^-,-^ | 0.221 | (-0.111, 0.554) |
|  | Average ^-,-^ | -0.058 | (-0.250, 0.133) | Average ^-,-^ | -0.039 | (-0.229, 0.151) |
|  | Good ^-,-^ | -0.024 | (-0.215, 0.167) | Good ^-,-^ | -0.034 | (-0.224, 0.156) |
| **IFIS**  **speed-agility** | Poor ^-,G^ | 0.250 | (0.015, 0.485) | Poor ^-,G^ | 0.233 | (-0.002, 0.468) |
|  | Average ^-,-^ | 0.006 | (-0.172, 0.185) | Average ^-,-^ | 0.018 | (-0.160, 0.196) |
|  | Good ^P,-^ | -0.291 | (-0.539, -0.042) | Good ^P,-^ | -0.295 | (-0.541, -0.048) |
| **IFIS**  **flexibility** | Poor ^-,G^ | 0.241 | (0.021, 0.461) | Poor ^-,G^ | 0.248 | (0.030, 0.466) |
|  | Average ^-,G^ | 0.013 | (-0.166, 0.192) | Average ^-,G^ | 0.016 | (-0.161, 0.193) |
|  | Good ^P,A^ | -0.372 | (-0.635, -0.110) | Good ^P,A^ | -0.389 | (-0.649, -0.129) |
| **Hs-CRP (Z-score)** | | | | | | |
| **IFIS**  **overall fitness** | Poor ^-,-^ | 0.133 | (-0.187, 0.453) | Poor ^-,-^ | 0.104 | (-0.220, 0.428) |
|  | Average ^-,G^ | 0.143 | (-0.027, 0.313) | Average ^-,G^ | 0.142 | (-0.028, 0.312) |
|  | Good ^-,A^ | -0.209 | (-0.392, -0.026) | Good ^-,A^ | -0.199 | (-0.383, -0.014) |
| **IFIS cardiorespiratory fitness** | Poor ^-,G^ | 0.176 | (0.013, 0.339) | Poor ^-,G^ | 0.154 | (-0.011, 0.320) |
|  | Average ^-,-^ | -0.058 | (-0.249, 0.133) | Average ^-,-^ | -0.035 | (-0.228, 0.159) |
|  | Good ^P,-^ | -0.481 | (-0.789, -0.172) | Good ^P,-^ | -0.463 | (-0.772, -0.154) |
| **IFIS**  **muscular fitness** | Poor ^-,-^ | 0.237 | (-0.069, 0.542) | Poor ^-,-^ | 0.203 | (-0.103, 0.510) |
|  | Average ^-,-^ | 0.057 | (-0.120, 0.234) | Average ^-,-^ | 0.067 | (-0.110, 0.244) |
|  | Good ^-,-^ | -0.141 | (-0.321, 0.039) | Good ^-,-^ | -0.140 | (-0.319, 0.040) |
| **IFIS**  **speed-agility** | Poor ^A,G^ | 0.299 | (0.090, 0.508) | Poor ^A,G^ | 0.313 | (0.102, 0.524) |
|  | Average ^P,-^ | -0.057 | (-0.225, 0.111) | Average ^P,-^ | -0.062 | (-0.230, 0.106) |
|  | Good ^P,-^ | -0.279 | (-0.520, -0.038) | Good ^P,-^ | -0.288 | (-0.527, -0.048) |
| **IFIS**  **flexibility** | Poor ^-,G^ | 0.241 | (0.043, 0.439) | Poor ^-,G^ | 0.257 | (0.059, 0.455) |
|  | Average ^-,-^ | -0.025 | (-0.194, 0.144) | Average ^-,-^ | -0.027 | (-0.195, 0.141) |
|  | Good ^P,-^ | -0.357 | (-0.617, -0.097) | Good ^P,-^ | -0.379 | (-0.638, -0.120) |
| Data are presented as mean and 95% confidence intervals. Adjusted models are adjusted by age and sex. Superscripts indicate statistically significant Tukey’s pairwise comparisons (p<0.05) for the Z-scores of cardiometabolic outcomes across categories of the IFIS scores: P (Poor), A (Average), and G (Good). For example, in IFIS overall fitness, for the unadjusted model, those rating their overall fitness as “Poor” had significant differences in the Z-score for cardiovascular health compared to those rating their overall fitness as “Good”.  CI: confidence interval, hs-CRP: high-sensitivity C-reactive protein, IFIS: International Fitness Scale. | | | | | | |
