# Supplementary material for: Validity of the International Fitness Scale (IFIS) and its associations with cardiometabolic health and body composition in adults with type 2 diabetes: A cross-sectional study
Source: PLoS One. 2026 Jan 6;21(1):e0339364. doi: 10.1371/journal.pone.0339364 (PMC12774367; doi:10.1371/journal.pone.0339364)
Supplement: S6 Table — Data are presented as mean and 95% confidence intervals. Adjusted models are adjusted by age and sex. Superscripts indicate statistically significant Tukey’s pairwise comparisons (p < 0.05) for the Z-scores of body composition outcomes across categories of the IFIS scores: P (Poor), A (Average), and G (Good). For example, in IFIS overall fitness, for the unadjusted model, those rating their overall fitness as “Poor” had significant differences in the Z-score for body mass index compared to those rating their overall fitness as “Average” or “Good”. CI: confidence interval, IFIS: International Fitness Scale. (DOCX) [file pone.0339364.s014.docx]

| **S6 Table . Differences in** **body composition outcomes (Z-scores) according to categories of self-reported (IFIS) physical fitness scores.** | | | | | | |
| --- | --- | --- | --- | --- | --- | --- |
|  | **Unadjusted** | | | | **Adjusted** | |
|  | **Category** | **Mean** | **95% CI** | **Category** | **Mean** | **95% CI** |
| **Body mass index (Z-score)** | | | | | | |
| **IFIS**  **overall fitness** | Poor ^A,G^ | 0.731 | (0.429, 1.033) | Poor ^A,G^ | 0.628 | (0.328, 0.928) |
|  | Average ^P,G^ | 0.165 | (0.005, 0.325) | Average ^P,G^ | 0.153 | (-0.004, 0.309) |
|  | Good ^P,A^ | -0.435 | (-0.609, -0.261) | Good ^P,A^ | -0.387 | (-0.558, -0.216) |
| **IFIS cardiorespiratory fitness** | Poor ^A,G^ | 0.286 | (0.124, 0.447) | Poor ^A,G^ | 0.248 | (0.088, 0.407) |
|  | Average ^P,-^ | -0.215 | (-0.403, -0.028) | Average ^P,-^ | -0.174 | (-0.358, 0.011) |
|  | Good ^P,-^ | -0.475 | (-0.789, -0.160) | Good ^P,-^ | -0.448 | (-0.754, -0.143) |
| **IFIS**  **muscular fitness** | Poor ^-,-^ | 0.065 | (-0.243, 0.373) | Poor ^-,-^ | 0.027 | (-0.271, 0.324) |
|  | Average ^-,-^ | -0.072 | (-0.255, 0.111) | Average ^-,-^ | -0.049 | (-0.225, 0.127) |
|  | Good ^-,-^ | 0.049 | (-0.134, 0.232) | Good ^-,-^ | 0.039 | (-0.136, 0.215) |
| **IFIS**  **speed-agility** | Poor ^A,G^ | 0.461 | (0.260, 0.661) | Poor ^A,G^ | 0.399 | (0.203, 0.596) |
|  | Average ^P,G^ | -0.072 | (-0.238, 0.094) | Average ^P,G^ | -0.032 | (-0.193, 0.129) |
|  | Good ^P,A^ | -0.478 | (-0.712, -0.245) | Good ^P,A^ | -0.475 | (-0.701, -0.250) |
| **IFIS**  **flexibility** | Poor ^A,G^ | 0.337 | (0.146, 0.529) | Poor ^-,G^ | 0.309 | (0.124, 0.493) |
|  | Average ^P,G^ | 0.011 | (-0.154, 0.177) | Average ^-,G^ | 0.037 | (-0.122, 0.196) |
|  | Good ^P,A^ | -0.625 | (-0.880, -0.369) | Good ^P,A^ | -0.635 | (-0.880, -0.391) |
| **Visceral adipose volume (Z-score)** | | | | | | |
| **IFIS**  **overall fitness** | Poor ^-,G^ | 0.560 | (0.228, 0.892) | Poor ^A,G^ | 0.650 | (0.348, 0.953) |
|  | Average ^-,G^ | 0.159 | (-0.013, 0.331) | Average ^P,G^ | 0.179 | (0.024, 0.334) |
|  | Good ^P,A^ | -0.335 | (-0.514, -0.156) | Good ^P,A^ | -0.383 | (-0.545, -0.220) |
| **IFIS cardiorespiratory fitness** | Poor ^-,G^ | 0.064 | (-0.108, 0.237) | Poor ^-,G^ | 0.176 | (0.015, 0.336) |
|  | Average ^-,G^ | 0.108 | (-0.089, 0.306) | Average ^-,G^ | -0.008 | (-0.191, 0.176) |
|  | Good ^P,A^ | -0.473 | (-0.784, -0.163) | Good ^P,A^ | -0.549 | (-0.832, -0.265) |
| **IFIS**  **muscular fitness** | Poor ^-,-^ | 0.339 | (0.017, 0.661) | Poor ^A,G^ | 0.438 | (0.142, 0.735) |
|  | Average ^-,-^ | -0.051 | (-0.237, 0.135) | Average ^P,-^ | -0.073 | (-0.244, 0.097) |
|  | Good ^-,-^ | -0.061 | (-0.245, 0.123) | Good ^P,-^ | -0.071 | (-0.241, 0.098) |
| **IFIS**  **speed-agility** | Poor ^A,G^ | 0.526 | (0.313, 0.739) | Poor ^A,G^ | 0.494 | (0.295, 0.693) |
|  | Average ^P,-^ | -0.102 | (-0.268, 0.064) | Average ^P,-^ | -0.096 | (-0.249, 0.058) |
|  | Good ^P,-^ | -0.432 | (-0.667, -0.198) | Good ^P,-^ | -0.407 | (-0.624, -0.190) |
| **IFIS**  **flexibility** | Poor ^-,-^ | 0.420 | (0.217, 0.624) | Poor ^A,G^ | 0.375 | (0.185, 0.566) |
|  | Average ^-,G^ | -0.045 | (-0.213, 0.122) | Average ^P,G^ | -0.043 | (-0.199, 0.113) |
|  | Good ^-,A^ | -0.533 | (-0.784, -0.283) | Good ^P,A^ | -0.470 | (-0.704, -0.236) |
| **Muscle volume (Z-score)** | | | | | | |
| **IFIS**  **overall fitness** | Poor ^-,-^ | -0.052 | (-0.399, 0.295) | Poor ^-,G^ | -0.231 | (-0.546, 0.085) |
|  | Average ^-,-^ | -0.115 | (-0.297, 0.066) | Average ^-,G^ | -0.146 | (-0.310, 0.017) |
|  | Good ^-,-^ | 0.139 | (-0.049, 0.327) | Good ^P,A^ | 0.225 | (0.054, 0.395) |
| **IFIS cardiorespiratory fitness** | Poor ^-,-^ | -0.023 | (-0.199, 0.152) | Poor ^-,G^ | -0.130 | (-0.293, 0.032) |
|  | Average ^-,-^ | -0.088 | (-0.290, 0.115) | Average ^-,-^ | 0.033 | (-0.153, 0.220) |
|  | Good ^-,-^ | 0.286 | (-0.028, 0.600) | Good ^P,-^ | 0.337 | (0.052, 0.623) |
| **IFIS**  **muscular fitness** | Poor ^-,G^ | -0.502 | (-0.816, -0.188) | Poor ^A,G^ | -0.566 | (-0.851, -0.281) |
|  | Average ^-,G^ | -0.090 | (-0.272, 0.092) | Average ^P,G^ | -0.055 | (-0.220, 0.110) |
|  | Good ^P,A^ | 0.257 | (0.076, 0.438) | Good ^P,A^ | 0.243 | (0.079, 0.407) |
| **IFIS**  **speed-agility** | Poor ^-,-^ | -0.160 | (-0.388, 0.067) | Poor ^-,G^ | -0.217 | (-0.426, -0.008) |
|  | Average ^-,-^ | -0.008 | (-0.185, 0.170) | Average ^-,-^ | 0.034 | (-0.128, 0.196) |
|  | Good ^-,-^ | 0.206 | (-0.043, 0.455) | Good ^P,-^ | 0.192 | (-0.035, 0.418) |
| **IFIS**  **flexibility** | Poor ^-,G^ | -0.184 | (-0.401, 0.033) | Poor ^-,G^ | -0.185 | (-0.384, 0.014) |
|  | Average ^-,-^ | 0.007 | (-0.170, 0.183) | Average ^-,-^ | 0.026 | (-0.136, 0.187) |
|  | Good ^P,-^ | 0.257 | (-0.006, 0.520) | Good ^P,-^ | 0.216 | (-0.026, 0.457) |
| Data are presented as mean and 95% confidence intervals. Adjusted models are adjusted by age and sex. Superscripts indicate statistically significant Tukey’s pairwise comparisons (p<0.05) for the Z-scores of body composition outcomes across categories of the IFIS scores: P (Poor), A (Average), and G (Good). For example, in IFIS overall fitness, for the unadjusted model, those rating their overall fitness as “Poor” had significant differences in the Z-score for body mass index compared to those rating their overall fitness as “Average” or “Good”.  CI: confidence interval, IFIS: International Fitness Scale. | | | | | | |
