# Supplementary material for: Validity of the International Fitness Scale (IFIS) and its associations with cardiometabolic health and body composition in adults with type 2 diabetes: A cross-sectional study
Source: PLoS One. 2026 Jan 6;21(1):e0339364. doi: 10.1371/journal.pone.0339364 (PMC12774367; doi:10.1371/journal.pone.0339364)
Supplement: Attachment — Submitted filename: Response to Reviewers.docx [file pone.0339364.s016.docx]

Note: line numbers in the present revision refer to the Manuscript with Track Changes. In it, modifications are highlighted in yellow, while deleted paragraphs are highlighted in red.

**REVIEWERS' COMMENTS:**

**Reviewer #1:**

**Herraiz-Adillo and colleagues aim to compare the international fitness scale (IFIS) that rests on patient’s ratings of their performance in five domains to the 6-minute walk test that is a submaximal exercise test providing a distance in meters. Further, Herraiz-Adillo and colleagues compare IFS and 6-min walk test in terms of cardiometabolic and body composition outcomes as assessed by visceral adipose tissue volume, high sensitive C reactive protein, tight muscle volume and hepatic triglyceride concentration.**

**Herraiz-Adillo and colleagues conclude that IFIS is a valid tool for assessing physical fitness in diabetic patients and that it displays cardiometabolic and body compositions outcomes at least**

**as well as the 6-minute walk test.**

Authors, general comment:

We would like to sincerely thank the reviewer for their thoughtful and constructive comments, which have contributed to improving the clarity and quality of our manuscript. As the manuscript is already quite lengthy, we have aimed to incorporate revisions as concisely as possible, while still ensuring that all key points are adequately addressed. In instances where comments were more general or lacked specific detail, we interpreted them as accurately as possible and made revisions accordingly.

**Major Comments:**

**1.1 Unless patients have serious cognitive impairment, one expects overall fitness and cardiorespiratory fitness assessments (from very poor, poor, average, good and very good) to somewhat correlate with walking distance over 6 minutes. (Fig 2)**

Authors:

Thank you for the comment. While a correlation between self-reported fitness (IFIS) and 6-minute walk test (6MWT) performance is intuitively expected in individuals without cognitive impairment, this relationship had not previously been demonstrated in patients with type 2 diabetes. Addressing this gap was a key rationale for our study.

In the EPSOMIP protocol (PMID: 33879084), cognitive status was not considered an exclusion criterion, making it unclear whether cognitive impairment may have influenced the results. The potential influence of response bias in the IFIS (potentially linked to cognitive impairment) has now been addressed in the article.

Regarding Figure 2, we believe it is central to evaluating the criterion validity of the IFIS. The figure depicts a clear, graded increase in mean 6MWT distance across IFIS categories, with ANCOVA adjusted for age and sex confirming statistically significant differences between levels. Moreover, our analytical approach aligns with that of the original validation study of the IFIS (PMID: 21441238), which also employed ANCOVA to examine criterion validity.

Revised version:

In Discussion, lines 506-508: “As a self-reported tool, the IFIS is subject to response bias, potentially influenced by cognitive impairment, which could distort its association with objective health markers.”

**1.2 While one expects the 6-minute walking distance to be somewhat indirectly related to body composition, the 6-minute walking test is not an appropriate tool to estimate body composition.**

Authors:

We appreciate the reviewer’s thoughtful observation. We fully agree that the 6MWT is not designed to estimate body composition and acknowledge its limitations in this context. These limitations were partially addressed in the original manuscript and have now been rewritten for a better flow with additional paragraphs (lines 505-508).

Apart from evaluating criterion validity, a key objective of our study is to evaluate the construct validity of the IFIS by analysing associations between self-reported fitness (via IFIS) and body composition parameters. This approach is consistent with the methodology used in the seminal IFIS validation study (PMID: 21441238), where similar associations supported construct validity.

Therefore, our intention was not to use the 6MWT as a proxy for body composition, but rather to explore the relationships between the IFIS and objective body composition measures as part of a broader assessment of its construct validity. The ANCOVA analyses presented in Supplementary Figure S3 (now Supplementary Figure S4), examining associations between 6MWT and body composition parameters, were just included as an indirect comparison, intended to contextualize how IFIS versus 6MWT relates to body composition.

This has been now clarified in the manuscript in Results.

Revised version:

In Discussion, lines 509-512: “We did not compare IFIS scores to objective tests of muscular fitness, speed-agility, or flexibility, limiting the breadth of criterion comparisons. However, cardiorespiratory fitness, the component analyzed in greater depth in our study, remains the aspect of physical fitness most consistently associated with health outcomes.[13]”

In Results, lines 347-351: “Both IFIS scores showed significant associations with Z-scores of cardiometabolic and, particularly, body composition outcomes (p<0.05 for ANCOVA in all comparisons). In general, better IFIS scores were associated with higher cardiovascular health scores and muscle volume, alongside lower liver fat content, high-sensitivity C-reactive protein, BMI, and visceral adipose volume. To provide context, we also examined associations of the 6-minute walk test with the same outcomes (see S4 Fig), which were broadly similar but not significant for liver fat or high-sensitivity C-reactive protein (p>0.05). These analyses were not intended to position the 6-minute walk test as a body composition measure, but rather to contextualize the construct validity.”

**Minor Comments:**

**1.3 The study population is relatively small (282 subjects). Cardiorespiratory fitness depends on the daily activities of patients. Whether they were sedentary or physically active due to their occupations is not mentioned. Comparing physical performance ratings by an active security guard and an office worker to distances walked over 6 minutes may not be valid.**

Authors:

Thank you for the insightful comment. While physical fitness is influenced by genetic factors, we agree that occupational activity—primarily through its impact on physical activity—can also contribute to physical and cardiorespiratory fitness.

In our dataset, we captured moderate-to-vigorous physical activity (MVPA) using 1–5 minute activity bouts recorded per day. We have now examined the interaction between IFIS scores and MVPA in predicting 6-minute walk test performance. Neither MVPA nor the IFIS × MVPA interaction terms were statistically significant (all P values >0.05), suggesting no clear evidence that the relationship between IFIS and walking distance varies by MVPA levels. So, we think that an analysis stratifying by MVPA is not warranted.

Moreover, we consider actual cardiorespiratory fitness (as measured by the 6MWT) to be the primary determinant of perceived fitness (as measured by the IFIS), rather than physical activity itself. In this conceptual framework, a corresponding directed acyclic graph (DAG) would position physical activity as an upstream determinant of cardiorespiratory fitness, and consequently of perceived fitness (see DAG 1). Therefore, adjusting for physical activity is not advisable, as it would not control for a true confounder, but rather for an ancestor in the causal pathway. Such adjustment could result in overadjustment, attenuating the association we aim to validate.

That said, if one assumes that physical activity also independently influences perceived fitness, not solely via improved cardiorespiratory capacity, then physical activity might act as a confounder (see DAG 2). However, our primary aim is not to establish a causal effect of 6MWT on IFIS, but to evaluate the criterion validity of IFIS as a practical proxy for cardiorespiratory fitness, an attribute shaped in part by physical activity. In this context, adjusting for physical activity would isolate only the portion of the 6MWT-IFIS relationship that is independent of physical activity (direct effect), thereby compromising the real-world construct we aim to assess.

**DAG 1	DAG 2**

Our analytical approach is consistent with the methodology used in the seminal IFIS validation study (PMID: 21441238).

To further clarify this point, we have now included the descriptive characteristics of participants by physical activity in Table 1 and S1 Table, allowing readers to better understand its distribution within the sample and its potential role in shaping fitness perception and performance. In addition, to answer other Reviewers’ comments, a paragraph about causal inference has been included in the Discussion.

Revised version:

In Discussion, lines 495-502:” Several limitations should be acknowledged. Although the cross-sectional design is appropriate for validation studies, enabling simultaneous assessment of the index test (IFIS) and reference standards, it inherently limits causal inference. Additionally, unmeasured confounders such as T2DM duration and treatment, diet, physical activity, socioeconomic status, and comorbidities may have influenced the observed associations. However, our objective was not to infer causality but rather to assess how well IFIS scores align with the 6-minute walk test and established cardiometabolic and body composition markers, supporting its construct and criterion validity in individuals with T2DM”.

Table 1 and S1 Table.

**1.4 Patients with cardiac or pulmonary diseases are instructed to walk at a speed that they can sustain (submaximal test) and as much as possible to avoid any stops. The maximal heart rate is not an endpoint unless for comparison of multiple 6-minute walk tests.**

Authors:

Thanks for the comment. We fully agree that the 6MWT is not designed to assess maximal effort and that heart rate is not typically used as a core outcome.

However, in our manuscript, we explicitly acknowledge that the 6MWT is a submaximal functional test, specifically: “ Cardiorespiratory fitness was objectively evaluated using the 6-minute walk test, a submaximal exercise test that measures the distance walked in meters over six minutes[8,12] with proven validity across different populations.[13,14]”

In addition, heart rate was not used as an endpoint but rather incorporated into a sensitivity analysis to evaluate whether very low exertion levels (i.e., post-test heart rate <60% of predicted maximum) may have influenced the observed associations. As noted in the manuscript: “A sensitivity analysis excluding those participants with a heart rate after the 6-minute test below 60% of the predicted maximal heart rate (n = 79) slightly attenuated the associations but did not alter the conclusions (S2 Fig).” This approach was intended to ensure robustness of our findings by verifying that the results were not driven by individuals with potentially low test engagement or exertion.

**1.5 Skeletal muscle intermuscular adipose tissue may have been a more sensitive endpoint that skeletal muscle volume (Miller Eur Heart J 2025)**

Authors:

Thanks for sharing this comment and such an interesting paper. In our main analysis, we prioritized skeletal muscle volume as the primary muscle-related variable based on its well-established role as a cardiometabolic risk factor. While muscle fat infiltration (as a proxy for intermuscular adipose tissue, IMAT) is a promising emerging marker of muscle quality, its prognostic value remains less established. Notably, in the recent study by Miller et al. (PMID: 40159388), skeletal muscle volume was significantly and independently associated with death and myocardial infarction, whereas IMAT volume showed only a weak association, and IMAT density, though predictive, is not yet widely adopted in clinical models. Given these considerations, and the fact that results related to muscle fat infiltration are broadly comparable to those for muscle volume (see graphics below), we prefer to retain muscle volume as the variable of interest.

**1.6 In brief the data provided by Herraiz-Adillo and colleagues may be of interest to epidemiologists in need of fitness data and of lesser interest to physicians taking care of diabetic patients.**

Authors:

Once again, thanks a lot for the review. The potential interest of the paper, considering also additional reviewers´ questions, has been now expanded in the Conclusion section.

Revised version:

In Conclusion, lines 533-548:

“Our findings extend previous research by demonstrating that the IFIS is a valid and feasible tool for assessing physical fitness in adults not only at low risk of CVD,[9,10,38,41–44] but also at high risk, such as adults with T2DM. In addition, IFIS overall and cardiorespiratory fitness scores showed associations with cardiometabolic risk and body composition outcomes at least as good as those observed with the 6-minute walk test.

While the IFIS showed limited ability to discriminate reduced functional capacity, it nonetheless offers an accessible and practical tool for evaluating physical fitness and cardiometabolic risk in adults with T2DM. This is particularly relevant in settings such as primary care, where objective fitness assessments may be difficult to implement.

For example, current American Diabetes Association (ADA) Standards of Care recommend assessing physical activity and fitness as part of a comprehensive lifestyle management strategy, given their close link to improved glycemic control, reduced cardiovascular risk, and better treatment adherence.[47,48] While IFIS is not specifically endorsed by the ADA, incorporating brief, validated tools such as the IFIS could help identify patients with low fitness levels who may benefit from targeted physical activity interventions, potentially improving T2DM outcomes.”

**Reviewer #2:**

**I am grateful for the opportunity to review this manuscript titled "Self-Reported Fitness (IFIS) in Adults with Type 2 Diabetes Mellitus: Validity and Associations with Cardiometabolic and Body Composition Outcomes." The study aims to evaluate the validity of the International Fitness Scale (IFIS) in assessing cardiorespiratory fitness compared to the 6-minute walk test and to explore their associations with cardiometabolic and body composition outcomes in adults with type 2 diabetes mellitus (T2DM), using a cross-sectional analysis of data from the EPSOMIP study.**

Authors, general comment:

We sincerely thank the reviewer for their thoughtful and exceptionally detailed comments, which have greatly contributed to enhancing the clarity and quality of our manuscript. We have carefully addressed all suggestions to the best of our ability. As the manuscript is already quite lengthy, we have aimed to incorporate revisions as concisely as possible, while still ensuring that all key points are adequately addressed. We hope that our responses and the corresponding changes meet the reviewer’s expectations.

**Title:**

**2.1 The title does not specify the study design as cross-sectional, which may mislead readers about the nature of the research, particularly since validity and associations could imply a longitudinal or experimental approach.**

Authors:

Thanks for the comment. Reviewed as suggested.

Revised version:

Title: “Validity of the International Fitness Scale (IFIS) and Its Associations With Cardiometabolic Health and Body Composition in Adults With Type 2 Diabetes: A Cross-Sectional Study”

**2.2 The title mentions "validity" and "associations" but omits explicit reference to the comparison between self-reported fitness (IFIS) and an objective measure (6-minute walk test), which is a central focus of the study, thus lacking clarity on its primary intent.**

Authors:

Thank you for the comment. While we agree that including the objective measure of fitness—the 6-minute walk test—is informative, we believe that incorporating it into the title makes it overly lengthy and less readable. Instead, we suggest highlighting the associations with body composition and cardiometabolic health, which we consider more central to the study’s broader implications. That said, we remain open to further discussion and are happy to revise the title should the editors consider it necessary.

Revised version:

Title: “Validity of the International Fitness Scale (IFIS) and Its Associations With Cardiometabolic Health and Body Composition in Adults With Type 2 Diabetes: A Cross-Sectional Study”

**Abstract:**

**2.3 The abstract states the aim "to assess the validity of the International Fitness Scale (IFIS) for evaluating cardiorespiratory fitness compared to the 6-minute walk test," but fails to specify that this refers to criterion validity, leaving an important methodological distinction unclear.**

Authors:

Thank you for the comment. We are not entirely sure we understand the concern, as the previous version of the manuscript explicitly stated: “To assess the validity of the International Fitness Scale (IFIS) for evaluating cardiorespiratory fitness compared to the 6-minute walk test (criterion validity) and to examine their associations with cardiometabolic and body composition outcomes (construct validity) in adults with type 2 diabetes mellitus (T2DM).” Given this, we believe the information is already appropriately included. However, we are opened to further clarify this section if the reviewer feels it is still unclear.

**2.4 The results claim "strong correlations" between IFIS scores and the 6-minute walk test, yet no correlation coefficients or p-values are provided, rendering the strength of these associations vague and unsubstantiated within the abstract.**

Authors:

Thank you for the comment. The previous version of the manuscript stated: “Both IFIS overall and cardiorespiratory fitness strongly correlated with performance in the 6-minute walk test (p < 0.001 for ANCOVA models)...” Thus, it already includes the statistical significance of the ANCOVA models. We chose not to use Pearson correlation because IFIS is an ordinal scale with five categories, which do not guarantee equal intervals between values. Applying Pearson correlation assumes linearity and interval-level measurement, which may not hold in this case. Instead, we used ANCOVA, which is more appropriate for ordinal predictors and avoids potential misinterpretation of the association. Our approach is also consistent with the methodology used in the seminal IFIS validation study (PMID: 21441238). Nevertheless, we have revised the sentence to avoid the phrase 'strong correlation,' as it might suggest Pearson correlations rather than ANOVAs. In addition, to incorporate other Reviewer’s comment, we have included information about ROC curves directly comparing IFIS cardiorespiratory fitness with 6MWT (reference test).

Revised version:

In Abstract, lines 43-46: “Results: Both IFIS overall and IFIS cardiorespiratory showed graded associations with 6MWT (p<0.001 for ANCOVA models). For discriminating performance below the population-predicted mean 6MWT distance, IFIS cardiorespiratory showed an AUC of 0.615 (95% CI: 0.552-0.678).”

**2.5 The conclusion asserts that IFIS "conveys cardiometabolic and body composition outcomes at least as well as the 6-minute walk test," but this is not directly supported by the abstract’s results, which only discuss correlations and AUC comparisons, not a comprehensive equivalence in conveying outcomes.**

Authors:

Thank you for the comment. In the Results section of the abstract, and in response to the comments from other reviewers, we have placed greater emphasis on findings related to criterion validity rather than construct validity. We have also included the discriminative performance of the IFIS cardiorespiratory fitness item against the 6-minute walk test, as well as its sensitivity and specificity for diagnosing a clinically meaningful condition.

To keep the abstract within the 300-word limit, we have removed the AUC values comparing IFIS and the 6-minute walk test in predicting cardiometabolic outcomes, as this comparison is less central to the primary objectives of our study. Additionally, given the multiple components evaluated by IFIS (five fitness components) and the diverse range of outcomes (including various cardiometabolic and body composition indicators), a clear and comprehensive comparison of all results within the abstract was deemed impractical.

Lastly, we have revised the Conclusion section to reflect and incorporate the additional comments from Reviewers.

Revised version:

In Results, Abstract, lines 43-46: “Results: Both IFIS overall and IFIS cardiorespiratory showed graded associations with 6MWT (p<0.001 for ANCOVA models). For discriminating performance below the population-predicted mean 6MWT distance, IFIS cardiorespiratory showed an AUC of 0.615 (95% CI: 0.552-0.678).”

In Abstract, lines 53-58: “This study supports the International Fitness Scale (IFIS) may be a practical tool for adults with T2DM, showing evidence of criterion validity via graded association with 6MWT performance, and construct validity via significant associations with cardiometabolic and body composition outcomes. Although its ability to discriminate impaired functional capacity was limited, IFIS may serve as a feasible alternative for fitness assessment when objective testing is not possible in primary care.”

**Introduction**

**2.6 The introduction notes the "rising prevalence" of T2DM but provides no specific statistics beyond a vague reference to "more than 500 million people" (Line 53), lacking precision and context to justify the global health concern.**

Authors:

Thank you, revised as suggested.

Revised version:

In Introduction, lines 61-63: "Type 2 diabetes mellitus (T2DM) is a growing global health concern, affecting more than 500 million people.[1] Its prevalence is rising alarmingly, with the disease burden, measured in DALYs, having tripled over the past 30 years.[2]”

**2.7 The link between T2DM and lifestyle factors is mentioned, but there is no elaboration on how physical fitness specifically influences T2DM management or outcomes, weakening the rationale for focusing on fitness.**

Authors:

Thank you, revised as suggested.

Revised version:

In Introduction, lines 68-70: “Adequate physical fitness, particularly cardiorespiratory fitness, not only reduces the risk of developing T2DM [4,5] but also improves glycemic control, insulin sensitivity, and cardiovascular outcomes in those already diagnosed.[6]”

**2.8 The claim that cardiorespiratory fitness reduces T2DM risk lacks a direct citation at this point (references [4,5] appear later), and its relevance to patients already diagnosed with T2DM is not established.**

Authors:

Thanks for the comment. As suggested, we have included references for the two different sentences. In addition, the reference

*Crump C, Sundquist J, Winkleby MA, Sieh W, Sundquist K. Physical Fitness Among Swedish Military Conscripts and Long-Term Risk for Type 2 Diabetes Mellitus: A Cohort Study. Ann Intern Med. 2016;164:577–584. doi:10.7326/M15-2002*

has been replaced with

*Ballin M, Ahlqvist VH, Berglind D, Brunström M, Herraiz-Adillo A, Henriksson P, et al. Cardiorespiratory fitness in adolescence and risk of type 2 diabetes in late adulthood in one million Swedish men: nationwide sibling controlled cohort study. BMJ Med. 2025;4:e001313.*

Although both references analyze similar populations, the new reference additionally includes sibling-controlled cohort analyses, which better account for potential confounders and therefore provide less biased estimates.

Revised version:

In Introduction, lines 68-70: “Adequate physical fitness, particularly cardiorespiratory fitness, not only reduces the risk of developing T2DM [4–5] but also improves glycemic control, insulin sensitivity, and cardiovascular outcomes in those already diagnosed.[6]

4. Tarp J, Støle AP, Blond K, Grøntved A. Cardiorespiratory fitness, muscular strength and risk of type 2 diabetes: a systematic review and meta-analysis. Diabetologia. 2019;62: 1129–1142. doi:10.1007/s00125-019-4867-4

5. Ballin M, Ahlqvist VH, Berglind D, Brunström M, Herraiz-Adillo A, Henriksson P, et al. Cardiorespiratory fitness in adolescence and risk of type 2 diabetes in late adulthood in one million Swedish men: nationwide sibling controlled cohort study. BMJ Med. 2025;4: e001313. doi:10.1136/bmjmed-2024-001313

6. Kanaley JA, Colberg SR, Corcoran MH, Malin SK, Rodriguez NR, Crespo CJ, et al. Exercise/Physical Activity in Individuals with Type 2 Diabetes: A Consensus Statement from the American College of Sports Medicine. Med Sci Sports Exerc. 2022;54: 353–368. doi:10.1249/MSS.0000000000002800.

**2.9 The objectives are stated, but the manuscript does not justify why cardiorespiratory fitness is prioritized over other fitness components (e.g., muscular fitness), despite IFIS assessing multiple domains.**

Authors:

Thank you for your relevant comment. In our study, while the IFIS overall score reflects general fitness, we specifically prioritized the IFIS cardiorespiratory fitness component over other domains such as muscular fitness, speed-agility, and flexibility. For the analysis of criterion validity against the 6-minute walk test, comparing it with the IFIS cardiorespiratory fitness subscale is more appropriate, as both measures assess the construct of cardiorespiratory fitness. Additionally, we emphasized IFIS cardiorespiratory fitness because it is the component most strongly associated with cardiovascular disease and mortality.

Revised version:

In Introduction, lines 101-103: “In this study, particular emphasis was placed on the cardiorespiratory fitness component of the IFIS, as it reflects a construct similar to that assessed by the 6-minute walk test and is the component more strongly associated with CVD and mortality.[12]”

**Methods**

**Study Design:**

**2.10 The cross-sectional design is mentioned, but there is no justification for its suitability to assess validity and associations, particularly given the inability to establish causality, which is a critical limitation for these objectives.**

Authors:

Thanks for giving us the opportunity to clarify this relevant issue. In the context of a validation study, especially one focused on assessing criterion validity (e.g., how well the IFIS correlates with other measures of fitness), causal inference is not typically the goal, making a cross sectional study design appropriate. In fact, in STARD (STAndards for the Reporting of Diagnostic Accuracy Studies) guidelines, one important criterion for assessing study quality and risk of bias is the temporal proximity between the index test and the reference standard. Thus, mentioning causality in this setting can be misleading. This aspect has now been clarified in the limitations section.

Revised version:

In Discussion, lines 495-502: “Several limitations should be acknowledged. Although the cross-sectional design is appropriate for validation studies, enabling simultaneous assessment of the index test (IFIS) and reference standards, it inherently limits causal inference. Additionally, unmeasured confounders such as T2DM duration and treatment, diet, physical activity, socioeconomic status, and comorbidities may have influenced the observed associations. However, our objective was not to infer causality but rather to assess how well IFIS scores align with the 6-minute walk test and established cardiometabolic and body composition markers, supporting its construct and criterion validity in individuals with T2DM.”

**2.11 The recruitment period is provided, but the manuscript omits the total number of eligible participants and response rate, hindering assessment of selection bias and representativeness.**

Authors:

We acknowledge the reviewer’s concern regarding the absence of the total number of eligible participants and the response rate. Patients with T2DM in Östergötland County attending annual checkups at their primary healthcare center between 2019 and 2023 were eligible for inclusion and were invited to participate by their diabetes nurse or treating physician. Due to the coronavirus disease 2019 pandemic and associated slow recruitment, additional invitations were sent via letters to patients registered at primary healthcare centers.

Given the large number of healthcare centers involved and the dual recruitment approach, it was not feasible to maintain a complete record of the total number of eligible individuals or calculate an accurate response rate. This dual recruitment approach has now been clarified in the Methods section. We also acknowledge that this limitation introduces the possibility of selection bias, which we have now explicitly stated in the Limitations section of the manuscript.

Revised version:

In Methods, lines 115-120: “Recruitment took place in Östergötland County, Sweden, primarily through four primary healthcare centers in the cities of Norrköping and Linköping. Individuals with T2DM were invited to participate during their routine annual check-ups with their diabetes nurse or treating physician. Due to slower enrolment during the coronavirus disease 2019 pandemic, additional invitations were sent by letter to patients registered at participating primary healthcare centers. The recruitment period spanned from 03/04//2019 to 22/09/2023.”

In Discussion, lines 513-518: “Selection bias is a further consideration both from the initial participant recruitment and from missing data, as only 282 of 345 eligible participants had complete IFIS and 6-minute walk test results. Moreover, individuals with alcohol use disorder or high alcohol consumption were excluded based on screening questionnaires (2.3%), which may limit generalizability to this subgroup or reflect potential underreporting, given that this prevalence is unusually low for a Swedish population.”

**2.12 Inclusion criteria reference "current guidelines" for T2DM diagnosis, but these guidelines are not specified (e.g., ADA, WHO).**

Authors:

Thank you, revised as suggested.

Revised version:

In Methods, lines 121-122: “In the EPSOMIP protocol, inclusion criteria included a diagnosis of T2DM based on current guidelines[15] and an age range of 35–75 years.”

*15. Definition and diagnosis of diabetes mellitus and intermediate hyperglycemia: report of a WHO/IDF consultation. Geneva: World Health Organization; 2006.*

**2.13 Exclusion criteria include "contraindication for performing Magnetic Resonance Imaging," but specific contraindications (e.g., pacemakers, claustrophobia) are not detailed.**

Authors:

Thank you, revised as suggested.

Revised version:

In Methods, lines 124-125: “ …contraindication for performing Magnetic Resonance Imaging (MRI) (pacemaker, ferrous metal implants/fragments, claustrophobia, extreme obesity, and/or pregnancy)…”

**2.14 The sample size of 282 is reported, but the manuscript lacks a breakdown of exclusions by reason (beyond 18 withdrawals, Page 5, Line 106), obscuring the participant flow and potential biases.**

Authors:

The requested information is presented in detail in Figure 1, flowchart of the study. Thank you.

**Variables:**

**2.15 The IFIS is described, but its validation in Swedish or in adults with T2DM is not addressed, raising concerns about its cultural and clinical appropriateness for this population.**

Authors:

Thank you for the comment. The IFIS questionnaire used in our study was obtained directly from http://profith.ugr.es/IFIS, where it was originally developed and validated under the umbrella of the HELENA study in adolescents (original reference: PMID: 21441238). IFIS has also been validated in a Swedish population of women in early pregnancy (PMID: 34811456). To our knowledge, it has not yet been directly validated in a Swedish population with T2DM, which represents the specific research gap in our study. The previous Swedish validation has been included as a reference, and we have clarified this research gap accordingly in the revised manuscript.

Revised version:

In Methods, lines 159-161: “The Swedish version has been validated in women during early pregnancy,[10] but not, to our knowledge, in adults with T2DM, the specific gap this study explores.”

**2.16 The 6-minute walk test is presented as a measure of cardiorespiratory fitness, but its limitations (e.g., influence of motivation, comorbidities like neuropathy) in T2DM patients are not discussed.**

Authors:

Thank you for the comments. To address another reviewer’s concern, information on neuropathy and angiopathy has been added to Table 1, as these conditions may help contextualize potential influences on physical performance. Additionally, the criterion validity of the overall IFIS and IFIS cardiorespiratory fitness, with respect to the presence or absence of neuropathy or angiopathy, has been analysed in S3 Fig and S4 Table. Regarding motivation, it is worth noting that the sensitivity analysis excluding potentially submaximal tests yielded results consistent with the main analysis, suggesting that this concern did not substantially affect our findings.

Revised version:

In Methods, lines 263-267: “To explore the contribution of T2DM-related conditions, i.e., neuropathy and angiopathy, to the criterion validity of IFIS, a complementary analysis was conducted using adjusted ANCOVA models comparing the 6-minute walk test performance across IFIS overall and IFIS cardiorespiratory fitness categories between T2DM participants with and without neuropathy/angiopathy.”

In Results, lines 320-324: “IFIS cardiorespiratory fitness showed adequate criterion validity against the 6-minute walk test in participants both with neuropathy/angiopathy (p=0.004 for ANCOVA) and without these complications (p=0.001). However, when considering IFIS overall, a significant association with 6-minute walk test performance was observed only in participants without neuropathy/angiopathy (p=0.015), S3 Fig and S4 Table.”

In Discussion, lines 519-527: “While the 6-minute walk test is a validated and practical field measure, it may not fully capture maximal cardiorespiratory capacity as laboratory-based assessments do, and may be influenced by varying motivation levels. Nonetheless, sensitivity analyses excluding low-effort performances yielded consistent results, supporting the robustness of our findings.

Finally, common T2DM-related complications such as neuropathy or angiopathy may also influence 6-minute walk performance. Notably, while both IFIS cardiorespiratory and overall fitness demonstrated adequate criterion validity with the 6-minute walk test in participants without these conditions, only IFIS cardiorespiratory fitness showed adequate criterion validity in those with angiopathy or neuropathy.”

**Data Sources & Measurement:**

**2.17 The cardiovascular health score (Life’s Essential 8) is introduced, but the manuscript lacks details on measurement methods for each component (e.g., blood pressure, lipids) and specific scoring cutoffs.**

Authors:

Thanks for the relevant comment. The calculation of the Life’s Essential 8 Factor score has been clarified in Methods and Supporting Information (Appendix 1).

Revised version:

In Methods, lines 180-188: “Cardiovascular health was assessed using the Life’s Essential 8 score, as defined by the American Heart Association.[22] In the present study, we focused specifically on Life’s Essential 8 factors (BMI, blood lipids, blood pressure, and diabetes) rather than Life’s Essential 8 behaviors (diet, sleep, physical activity, and smoking), as factors more directly reflect cardiometabolic outcomes and are more likely to be influenced by physical fitness. The Life´s Essential 8 Factor score accounts for actual values of non-HDL cholesterol, blood pressure, and blood glucose, along with the use of medication for these factors, as well as values of BMI. A detailed description of the scoring methodology has been provided as Supporting Information (Appendix 1).”

In Supporting Information, Appendix 1.

**2.18 Liver fat measurement via PDFF is noted, but its accuracy or potential biases (e.g., in T2DM patients with altered liver metabolism) are not discussed, questioning its validity in this context.**

Authors:

Thank you for your comment. MR-PDFF is widely regarded as the gold standard for non-invasive liver fat quantification, with its technical performance, bias, and linearity extensively validated in the literature (PMID: 28892458; PMID: 22025886). Furthermore, MRS-PDFF demonstrates excellent correlation with the proportion of liver biopsy area involved by steatosis on histopathology (PMID: 28286210). In addition, we are not aware of any studies that have suggest potential bias in the PDFF performance in a T2DM population. We have now included an appropriate reference in the manuscript to support this statement.

Revised version:
In Methods, lines 195-197: “Liver fat (%) was quantified based on the hepatic triglyceride concentration, measured using PDFF by Magnetic Resonance Spectroscopy (MRS).[14,24]”

*14.* *Nasr P, Iredahl F, Dahlström N, Rådholm K, Henriksson P, Cedersund G, et al. Evaluating the prevalence and severity of NAFLD in primary care: the EPSONIP study protocol. BMC Gastroenterol. 2021;21: 180. doi:10.1186/s12876-021-01763-z*

*24. Nasr P, Forsgren MF, Ignatova S, Dahlström N, Cedersund G, Leinhard OD, et al. Using a 3% Proton Density Fat Fraction as a Cut-Off Value Increases Sensitivity of Detection of Hepatic Steatosis, Based on Results From Histopathology Analysis. Gastroenterology. 2017;153: 53-55.e7. doi:10.1053/j.gastro.2017.03.005*

**Bias & Confounding:**

**2.19 Adjustments for sex and age are mentioned in ANCOVA models, but other potential confounders (e.g., T2DM duration, medication use, physical activity levels) are not addressed.**

Authors:

Thanks for the comment. As has been addresses in comment 10, mentioning causality or confounding in this setting can be misleading. The reason is that our objective is not to infer causality but to assess the extent to which IFIS scores align with the 6-minte walk tests and established cardiometabolic and body composition markers, thereby supporting its construct and criterion validity in individuals with T2DM.

Revised version:

In Discussion, lines 495-502: “Several limitations should be acknowledged. Although the cross-sectional design is appropriate for validation studies, enabling simultaneous assessment of the index test (IFIS) and reference standards, it inherently limits causal inference. Additionally, unmeasured confounders such as T2DM duration and treatment, diet, physical activity, socioeconomic status, and comorbidities may have influenced the observed associations. However, our objective was not to infer causality but rather to assess how well IFIS scores align with the 6-minute walk test and established cardiometabolic and body composition markers, supporting its construct and criterion validity in individuals with T2DM.”

**2.20 ROC curve use is described, but the manuscript does not consider the risk of overfitting or the need for external validation.**

Authors:

We thank the reviewer for raising this important point. While our primary aim was to validate the International Fitness Scale (IFIS) rather than to develop or evaluate a predictive model, we acknowledge that ROC curve analyses, even in validation contexts, may yield biased estimates when applied within a single dataset. Although the risk of overfitting is minimal due to the simplicity of the IFIS instrument and the lack of model complexity, we agree that external validation in independent T2DM populations would strengthen the generalizability of our findings. We have now added this point to the discussion.

Revised version:

In Discussion, lines 437-440: “Future studies incorporating ROC analyses in independent cohorts are warranted to refine the discriminative performance of IFIS, improve its generalizability, and explore strategies to enhance the resolution of self-reported fitness measures.”

**Sample Size & Statistical Methods:**

**2.21 The sample size of 282 is stated, but no power calculation is provided to justify its adequacy for detecting differences or associations.**

Authors:

A detailed sample size calculation is available in the EPSOMIP protocol (PMID: 33879084). However, this calculation is not directly relevant to the current study, which focuses on an exploratory post hoc study within the EPSOMIP protocol. In the present article, we did not perform post hoc power calculations, as they are not considered informative according to current methodological guidance (PMID: 35642557).

**2.22 Statistical methods mention ANCOVA and ROC curves, but handling of missing data (e.g., for 247 participants with MASLD data vs. 282 total, Page 10, Line 234) is not explained.**

Authors:

Thank you for the comment. The flow chart of the study clarifies the final sample size considered for each outcome. To maximize statistical power, we did not restrict the final sample to participants with complete data across all outcomes; instead, we included all available data for each specific analysis.

Revised version:

In Methods, lines 268-270: “Analyses were restricted to participants with complete data on IFIS and the 6-minute walk test. For cardiometabolic and body composition outcomes, an available-case approach was used, including all participants with data available for each outcome”.

**Results**

**2.23 Table 1 stratifies characteristics by IFIS overall fitness, but the manuscript lacks a full distribution of participants across all IFIS categories (e.g., all five levels).**

Authors:

Thank you, revised as suggested.

Revised version:

In Methods, lines 276-277: “The characteristics of participants are presented in Table 1 (stratified by three IFIS overall fitness categories) and S1 Table (stratified by five categories).”

**2.24 Mean age and other characteristics are reported, but differences across IFIS categories are not statistically tested or discussed.**

Authors:
Thank you for your comment. In response, we have now included p-values to assess differences in the descriptive variables across IFIS categories in Table 1. For continuous variables, unadjusted ANOVA models were used, while for categorical variables, chi-square tests were applied.

However, it is important to note that this study was not originally powered to detect differences across these groups. Since statistical significance is highly dependent on sample size, the tests may be overly sensitive, potentially identifying statistically significant differences that are not clinically meaningful, or failing to detect meaningful differences.

Our primary aim in Table 1 was to provide a descriptive overview of participant characteristics across fitness categories, rather than to draw inferential conclusions about group differences.

**Outcome Data & Main Results:**

**2.25 Fig 2 shows dose-response associations, but specific statistical measures (e.g., regression coefficients) are not provided, obscuring the magnitude and precision of these relationships.**

Authors:

Thank you for the opportunity to clarify this point. Figure 2 illustrates the association between self-reported IFIS overall and cardiorespiratory fitness and objectively measured cardiorespiratory fitness via the 6-minute walk test, using ANCOVA models. P-values for these models are provided in the figure (both p < 0.001 for IFIS overall and IFIS cardiorespiratory fitness).

In addition, the specific means and 95% confidence intervals along with Tukey’s pairwise comparisons for the 6-minute walk test across IFIS strata—both unadjusted and adjusted—are reported in Supplementary Table S2. We believe these details sufficiently convey the magnitude and precision of the relationships.

We chose ANCOVA models over linear regression because the associations may not be strictly linear, and ANCOVA provides clinically interpretable comparisons across categorical IFIS groups. We believe this approach offers a more meaningful depiction of the relationship for the clinical and public health audience, and its also consistent with the seminal paper validating IFIS (PMID: 21441238).

**2.26 "Significant associations" are reported, but p-values and confidence intervals are absent in the text (only in figures).**

Authors:

Thank you for this comment. We believe the reviewer is referring to Figure 3. As noted, this figure includes p-values and measures of uncertainty (standard errors of the mean) for each estimate. Figure 3 presents the results from 12 comparisons (6 outcomes × 2 IFIS fitness domains). We sincerely believe that including all individual p-values and confidence intervals in the main text would be lengthy, potentially redundant, and could detract from the overall readability of the manuscript.

**2.27 ROC analysis results include AUCs, but their clinical significance (e.g., practical utility of AUC 0.678 for cardiovascular health) or implications are not addressed.**

Authors:

Thank you for raising this important point. We have now addressed the clinical interpretation of the AUC values in the Discussion section, which also helps respond to a related comment from another reviewer. Although the example provided refers to the comparison between IFIS and the 6-minute walk test, it helps clarify the practical utility and limitations of AUCs for IFIS in identifying various outcomes. In addition, a new table (S3 Table) has been added to illustrate the diagnostic performance of the IFIS cardiorespiratory fitness score in identifying clinically meaningful thresholds of functional capacity, as defined by the 6-minute walk test.

Revised version:

In Discussion lines 421-433: “Moreover, reporting less than “good” cardiorespiratory fitness (i.e., “very poor,” “poor,” or “average”) on the IFIS yielded sensitivities of 93.9% and 87.5%, and negative predictive values of 82.1% and 94.4% for identifying individuals with reduced performance on the 6-minute walk test, defined as walking below the median distance or achieving less than 80% of their individualized predicted distance, respectively. Although the AUCs indicated poor discriminative ability (approximately 0.600), this may reflect the ordinal nature of IFIS, participants’ tendency to avoid selecting the extreme categories (“very poor” or “very good”), and the considerable variation in 6-minute walk test distances among participants within the same IFIS category, rather than a true lack of association. The 6-minute walk test is also not a formal gold-standard test, which should be considered when interpreting these results. Nevertheless, the high sensitivity and negative predictive values suggest that IFIS may serve as a practical first-line screening tool to flag individuals for further assessment. “

In Supporting information, S3 Table.

**Discussion**

**2.28 The claim that IFIS is a "valid tool" is based on cross-sectional data, which cannot fully establish validity (e.g., predictive or longitudinal validity).**

Authors:

Thanks for the relevant comment. This issue has been addressed in comments 10 and 19.

**2.29 IFIS outperforming the 6-minute walk test for some outcomes is noted, but reasons (e.g., self-report bias, test limitations) or implications are not explored.**

Authors:

Thank you for the comment. Although speculative, we believe that any self-report bias in IFIS is likely non-differential across its five components. Therefore, the superior performance of IFIS overall fitness may be primarily due to its ability to capture multiple dimensions of fitness, whereas the 6-minute walk test mainly reflects aerobic capacity. The limitations of the 6-minute walk test are discussed in a separate paragraph.

Revised version:

In Discussion, lines 463-466: “In fact, we observed that the IFIS overall fitness score outperformed the 6-minute walk test to predict poor cardiovascular health and MALFD, possibly due to IFIS overall fitness capturing multiple fitness dimensions, while the 6-minute walk test mainly reflects aerobic capacity.”

**2.30 The lack of test-retest reliability is acknowledged, but other biases (e.g., self-report bias in IFIS, selection bias from MRI exclusions) are not discussed.**

Authors:

Thank you for raising this important point. In our study, 345 participants accepted the invitation to participate in the EPSOMIP study, and ultimately 282 had complete data for evaluating the IFIS against the 6-minute walk test. Some additional participants were excluded when analysing associations with body composition and cardiometabolic risk outcomes. This corresponds to a minimum missingness of 18.3%, which could be problematic if the data are missing not at random (MNAR).

Regarding MRI exclusions, only 4 participants (1.2%) from the invited sample were excluded due to contraindications (e.g., pacemakers, prostheses). While such exclusions could potentially reflect a more pathological subgroup indicating MNAR, the percentage is well below the commonly cited 5% threshold that typically raises concerns about selection bias.

Nevertheless, we agree that it is appropriate to acknowledge potential selection bias, particularly in relation to missing data and self-report bias inherent in the IFIS. However, we believe that MRI-related exclusions are unlikely to have introduced significant bias in our study due to their low frequency.

Revised version:

In Discussion, lines 506-508: “As a self-reported tool, the IFIS is subject to response bias, potentially influenced by cognitive impairment, which could distort its association with objective health markers.”
In Discussion, lines 513-518: “Selection bias is a further consideration both from the initial participant recruitment and from missing data, as only 282 of 345 eligible participants had complete IFIS and 6-minute walk test results. Moreover, individuals with alcohol use disorder or high alcohol consumption were excluded based on screening questionnaires (2.3%), which may limit generalizability to this subgroup or reflect potential underreporting, given that this prevalence is unusually low for a Swedish population.”

**2.31 The 6-minute walk test’s limitations as a proxy are mentioned, but its specific shortcomings in T2DM patients (e.g., neuropathy affecting walking) are not detailed.**

Authors:

Thank you for this relevant comment. Please, see comment 16.

**2.32 Previous IFIS research is referenced, but specific comparisons to studies in similar populations (e.g., adults with chronic conditions) are absent.**

Authors:

Thanks for raising this comment. Direct comparisons between our results and those of previous studies are challenging due to methodological differences, such as the use of ANCOVA models and variations in IFIS score categorization. Additionally, differences in study populations further limit comparability. However, we have revised the paragraph to include an indirect comparison with other studies in adult populations that also support the good criterion validity of IFIS, which is consistent with our findings.

Revised version:
In Discussion, lines 412-417: “After its original validation in adolescents,[9] the IFIS has demonstrated acceptable criterion validity against the 6-minute walk test in various adult populations, including women in early pregnancy,[10] women with fibromyalgia,[32] and older adults.[33] Our study expands this evidence to adults with T2DM. Consistent with previous findings, our study found that the IFIS cardiorespiratory score obtained a clear graded association with the 6-minute walk test.”

**2.33 The call for further studies is vague, lacking specific research questions or hypotheses (e.g., longitudinal validation, hard endpoints).**

Authors:

Thanks for the comment. As suggested, this paragraph has now been modified and located in the end of the Discussion, before Conclusions.

Revised version:

In Discussion, lines 528-531: “Further research is needed to assess the longitudinal validity of the IFIS and its predictive value for clinically relevant hard outcomes, including all-cause and CVD-related mortality and morbidity. Such efforts will help determine the utility of IFIS across broader settings and support its potential application in routine clinical decision-making.”

**Reviewer #3: Review Plos One june 2025**

**The study evaluates the coherence between a cardiorespiratory fitness questionnaire and the 6 min walking test, while also assesses associations to cardiometabolically associated variables. The study is based on a sample from the Swedish primary care, and is interesting, while providing new insights on IFIS. However, the manuscript would benefit from being more clear in precisely what has been analysed, what was found, and what it potentially means, while avoiding bold statements.**

Authors, general comment:

We would like to sincerely thank the reviewer for their thoughtful and constructive comments, which have contributed to improving the clarity and quality of our manuscript. As the manuscript is already quite lengthy, we have aimed to incorporate revisions as concisely as possible, while still ensuring that all key points are adequately addressed. We hope that our responses and the associated changes meet the reviewer’s expectations.

**Major:**

**3.1 Please clarify ”The American Heart Association recognizes cardiorespiratory fitness as a vital clinical sign”. How can a continous scale be considered a sign, especially of vitality? Are there established and valitated cutoffs which have clinical significance for specific tests and in what clinical settings are they used and for what purpose? This information should be considered also in the rest of the manuscript if the word ”clinical” is to be used. In general, the study would benefit from information on the validity of specific cutoffs, as this is often how tests are used in a clinical setting; ”good enough” or ”pathological”. If this questionnaire is currently used, how is it precisely used and to what benefit?**

Authors:

The American Heart Association (AHA) recognizes cardiorespiratory fitness (CRF) as a vital clinical sign due to strong evidence linking low CRF with higher risks of cardiovascular disease, all-cause mortality, and cancer-related deaths. Although CRF is measured on a continuous scale, values below 5 METs have consistently indicated increased mortality risk in different populations and clinical settings, while levels above 8–10 METs suggest better survival (PMID: 27881567). Additionally, a large meta-analysis in the general population found that each 1-MET increase in cardiorespiratory fitness—measured via maximal or submaximal exercise testing on a cycle ergometer or treadmill—was associated with an 11% reduction in mortality risk (PMID: 35562197).

In clinical practice, especially for healthy adults, the AHA recommends submaximal or field tests like the 6-minute walk test (6MWT) to estimate CRF. However, converting METs to 6MWT distance is challenging due to individual variability and test assumptions. Nevertheless, reference equations accounting for age, sex, height, and weight enable calculation of percent predicted 6MWT distance, which is a practical way to assess functional capacity (PMID: 9817683).

While no universal standard exists, a cutoff of <80% predicted 6MWT distance is commonly used, by analogy with other clinical tests, i.e. pulmonary tests, to indicate impaired functional capacity. This threshold helps identify patients needing further evaluation or intervention. Although not formally standardized, it is widely accepted in clinical and research settings. In addition, the influence of diabetes mellitus in the 6-min walk test has been explored (PMID: 30606177).

Note: In our study, the observed 6-minute walk test distance (538.9 meters) aligns with the predicted value (522.9 meters) and with results reported in other type 2 diabetes mellitus populations, such as the 520.8 meters reported by [PMID: 30606177].

As suggested, we have clearly defined cutoffs with clinical utility and revised the use of the term “clinical” throughout the manuscript. Additionally, in agreement with comment 5, we have estimated diagnostic performance measures based on the established clinical cutoffs (Table S3). In our study, despite demonstrating the criterion and construct validity of the IFIS, we acknowledged its limited discriminative ability as a diagnostic tool for identifying clinically meaningful cutoffs. Accordingly, we have softened claims in the conclusions to reflect these limitations.

Revised version:

In abstract, lines 53-58: “Conclusion: This study supports the International Fitness Scale (IFIS) may be a practical tool for adults with T2DM, showing evidence of criterion validity via graded association with 6MWT performance, and construct validity via significant associations with cardiometabolic and body composition outcomes. Although its ability to discriminate impaired functional capacity was limited, IFIS may serve as a feasible alternative for fitness assessment when objective testing is not possible in primary care.”

In Statistical analysis, lines 234-244: “To assess the diagnostic performance of the IFIS cardiorespiratory fitness item, we evaluated sensitivity, specificity, likelihood ratios, diagnostic odds ratios, and predictive values. Two thresholds were used to define reduced 6MWT performance:

i) a population-based threshold, defined as performance below the population-predicted mean 6-minute walk test distance (522.9 meters), calculated using the Enright & Sherrill equations based on the weighted average of age, height, and weight for men and women in the study population; and ii) an individualized threshold, also based on the Enright & Sherrill equations, which account for each participant’s age, sex, height, and weight. Functional impairment was defined as walking less than 80% of the individualized predicted distance.[17] Areas under the receiver operating characteristic (ROC) curves (AUCs) were calculated based on these thresholds to provide complementary insights into functional capacity.

In Results, lines 313-319: “To identify individuals with reduced 6-minute walk test performance, defined as either walking below the median on the 6-minute walk test or achieving less than 80% of their predicted distance, reporting “very poor”, “poor” or “average” cardiorespiratory fitness on the IFIS showed a sensitivity of 93.9% (95% CI: 87.8, 97.5) and specificity of 19.0% (95% CI: 13.4, 25.8) for the former, and a sensitivity of 87.5% (95% CI: 61.7, 98.4) and specificity of 13.3% (95% CI: 9.4, 18.1) for the latter. The corresponding AUCs were 0.615 (95% CI: 0.552, 0.678) and 0.556 (95% CI: 0.404–0.708), respectively (see S3 Table).”

In Discussion, lines 421-440: “Moreover, reporting less than “good” cardiorespiratory fitness (i.e., “very poor,” “poor,” or “average”) on the IFIS yielded sensitivities of 93.9% and 87.5%, and negative predictive values of 82.1% and 94.4% for identifying individuals with reduced performance on the 6-minute walk test, defined as walking below the median distance or achieving less than 80% of their individualized predicted distance, respectively. Although the AUCs indicated poor discriminative ability (approximately 0.600), this may reflect the ordinal nature of IFIS, participants’ tendency to avoid selecting the extreme categories (“very poor” or “very good”), and the considerable variation in 6-minute walk test distances among participants within the same IFIS category, rather than a true lack of association. The 6-minute walk test is also not a formal gold-standard test, which should be considered when interpreting these results. Nevertheless, the high sensitivity and negative predictive values suggest that IFIS may serve as a practical first-line screening tool to flag individuals for further assessment. To our knowledge, this is the first study to evaluate the discriminative performance of IFIS using ROC curves in adults with T2DM, providing clinically interpretable metrics for identifying individuals with reduced functional capacity. These findings support the criterion validity of the IFIS cardiorespiratory item while highlighting its limitations as a standalone diagnostic measure. Future studies incorporating ROC analyses in independent cohorts are warranted to refine the discriminative performance of IFIS, improve its generalizability, and explore strategies to enhance the resolution of self-reported fitness measures.”

**3.2 The authors argue that patients with ”conditions like peripheral neuropathy or peripheral arterial disease” may be unable to perform a physical assessment, which would indicate that this group may benefit from the FIS questionnaire instead. While these complications are common in T2D, it is very rare that they prevent a person from walking for 6 minutes. Furthermore, information on neuropathy and arterial disease in study participants is missing from this study. Therefore, this study does not validate the test for these individuals. Please rephrase the introduction as it is currently missleading. Also, please add the absence of this characterisation this to the limitation of the manuscript.**

Authors:

Thank you for these comments. In our database, we identified the variable “assessment of diabetic foot risk,” which has now been incorporated into Table 1 to better characterize our study population with respect to this important complication. Overall, 20.3% of participants presented with neuropathy or angiopathy, representing a sizable subgroup for validation purposes.

The potential impact of neuropathy and angiopathy on 6-minute walk test (6MWT) performance likely depends on the severity of these conditions. However, prior research has shown a consistent trend toward poorer performance in individuals with neuropathic T2DM compared to those without neuropathy, which was also confirmed in our study (mean 6MWT distance: 511.9 m vs. 550.7 m, p = 0.004), (PMID: 37116973).

We have now included a complementary analysis comparing IFIS overall and IFIS cardiorespiratory fitness scores with the 6MWT (criterion validity) regarding neuropathy/angiopathy, which we believe offers additional insights into the validity of IFIS in the context of T2DM and its complications. The introduction has been maintained, as it is now aligned with these new analyses.

Revised version:

In Methods, lines 263-267: “To explore the contribution of T2DM-related conditions, i.e., neuropathy and angiopathy, to the criterion validity of IFIS, a complementary analysis was conducted using adjusted ANCOVA models comparing the 6-minute walk test performance across IFIS overall and IFIS cardiorespiratory fitness categories between T2DM participants with and without neuropathy/angiopathy.”

In Results, lines 320-324: “IFIS cardiorespiratory fitness showed adequate criterion validity against the 6-minute walk test in participants both with neuropathy/angiopathy (p=0.004 for ANCOVA) and without these complications (p=0.001). However, when considering IFIS overall, a significant association with 6-minute walk test performance was observed only in participants without neuropathy/angiopathy (p=0.015), S3 Fig and S4 Table.”

In Discussion, lines 523-527: “Finally, common T2DM-related complications such as neuropathy or angiopathy may also influence 6-minute walk performance. Notably, while both IFIS cardiorespiratory and overall fitness demonstrated adequate criterion validity with the 6-minute walk test in participants without these conditions, only IFIS cardiorespiratory fitness showed adequate criterion validity in those with angiopathy or neuropathy.”

**3.3 It is problematic to discuss that the FIS-questionnaire ”conveys” cardiovascular risk based on the current study design with cross-sectional associations to established risk factors for cardiovascular disease. It is likely better for any clinician to simply measure blood pressure, LDL and glucose instead of using the questionnaire. Please rephrase this to reflect that you find that the FIS-questionnaire ”associates” with risk factors to a similar magnitude as the walking test.**

Authors:

Thank you for the insightful comment. As suggested, we have revised the manuscript to avoid language that may imply causality or overstate the findings. Throughout the text, we have replaced stronger verbs (e.g., "conveys") with more appropriate terms such as "associates," to accurately reflect the cross-sectional nature of our study.

**3.4 The study aims to ”validate” the IFIS questionnaire. However, it is not clearly stated what á priori defined criteria had to be met in order for the IFIS to be considered ”valid”. Please refer to general and established criteria for instrument validation which are typically used for validation of instruments in clinical use, and discuss your results according to effect sizes and approprietness of tests utilized in this study. Also, ”good criterion validity for assessing cardiorespiratory fitness when compared with objectively measured cardiorespiratory fitness tests, such as the 6-minute walk test” Please define what is ”good validity” is and how you concluded that the validity in this case was good.**

Authors:

Thank you for this constructive comment.

In our study, we assessed the validity of the IFIS questionnaire by examining both criterion and construct validity, following widely accepted standards for clinical instrument validation. For criterion validity, we compared IFIS scores to the 6-minute walk test (6MWT), an established objective measure of cardiorespiratory fitness. Initially, we used ANCOVA models based on prior foundational studies (PMID: 21441238). In response to your comment 5, we have now expanded our analysis to include standard diagnostic performance metrics, such as sensitivity, specificity, predictive values, likelihood ratios, and ROC curves, enabling a more rigorous and quantitative evaluation.

However, it is important to recognize that “good validity” for an instrument like IFIS cannot be reduced to a simple valid/invalid dichotomy. Validity encompasses multiple approaches (criterion and construct), various statistical estimators (ANCOVAs, ROC curves, likelihood ratios…), along different IFIS components, and a range of outcomes (body composition, cardiometabolic risk, etc.)

We have now included clarifying statements in the Discussion section that explicitly outline the criteria and thresholds used to interpret the validity of IFIS.

Revised version:
In Discussion, lines 421-440: “Moreover, reporting less than “good” cardiorespiratory fitness (i.e., “very poor,” “poor,” or “average”) on the IFIS yielded sensitivities of 93.9% and 87.5%, and negative predictive values of 82.1% and 94.4% for identifying individuals with reduced performance on the 6-minute walk test, defined as walking below the median distance or achieving less than 80% of their individualized predicted distance, respectively. Although the AUCs indicated poor discriminative ability (approximately 0.600), this may reflect the ordinal nature of IFIS, participants’ tendency to avoid selecting the extreme categories (“very poor” or “very good”), and the considerable variation in 6-minute walk test distances among participants within the same IFIS category, rather than a true lack of association. The 6-minute walk test is also not a formal gold-standard test, which should be considered when interpreting these results. Nevertheless, the high sensitivity and negative predictive values suggest that IFIS may serve as a practical first-line screening tool to flag individuals for further assessment. To our knowledge, this is the first study to evaluate the discriminative performance of IFIS using ROC curves in adults with T2DM, providing clinically interpretable metrics for identifying individuals with reduced functional capacity. These findings support the criterion validity of the IFIS cardiorespiratory item while highlighting its limitations as a standalone diagnostic measure. Future studies incorporating ROC analyses in independent cohorts are warranted to refine the discriminative performance of IFIS, improve its generalizability, and explore strategies to enhance the resolution of self-reported fitness measures.”

**3.5 If clinical validation is to be assessed, please consider to evaluate what sensitivity, specificity, PPV and NPV IFIS has to identify individuals with a poor performance of the 6 min walking test.**

Authors:

Thank you for this valuable comment. We agree that presenting the diagnostic performance of the IFIS is important for clinical interpretation. To address this, we have added Supplementary Table S3, which presents the diagnostic performance of the IFIS cardiorespiratory fitness score across two thresholds of functional capacity: <80% of the predicted 6-minute walk test (6MWT) distance, and below the population-predicted mean 6-MWT distance. Predicted distances were calculated using the Enright & Sherrill equations (PMID: 9817683), which account for sex, age, height, and weight. Although developed in healthy adults, these reference values (576 m for men and 494 m for women) align with those observed in our T2DM cohort (557 m for men and 505 m for women), supporting their relevance in this population.

Revised version:

Please, see comment 1.

**Minor:**

**3.6 The word ”proves” is used by the authors to state the valitidy of the quesitionnaire, which is a bit strong as a statement. While there is a correlation between the test and objective estimation of fitness in a selected group of patients with T2D in a single site observational study. That is not sufficient evidence to prove something. ”Indicates” may be more appropriate.**

Authors:

As suggested, the use of the verb “prove” has been corrected along the document to “indicate”. Thanks.

**3.7 The authors argue that the IFIS-questionnaire is easier to use than the 6-min walking test. Please clarify how long the FIS-test takes to complete to further support this claim.**

Authors:

Thank you, revised as suggested.

Revised version:

In Discussion, lines 477-480: “In this sense, along with the supported criterion and construct validity, IFIS offers a highly feasible approach as it only requires a single self-reported response that typically takes about 1-2 minutes to complete.[10]”

**3.8 Please communicate more clearly when it has meaning to objectively assess the cardiorespiratory fitness of a patient with T2D? Is this clinically used today or is this more of an instrument for academic purposes?**

Authors:

Thanks for the comment. Please, see comment 3.13.

**3.9 Patients with alcohol problems are excluded from the study. How was this defined? This may cause a problem to the external validity of the study, as 1/6 individuals in Sweden have a harmful use of alcohol.**

Authors:

In the EPSOMIP protocol (previously named EPSONIP), alcohol consumption was assessed using the validated AUDIT and Lifetime Drinking History (LDH) questionnaires. As shown in the study flow chart, 8 out of 345 participants (2.3%) were excluded due to high alcohol consumption or alcohol use disorder. Although this exclusion rate is lower than expected based on population estimates, we acknowledge that this may introduce selection bias, limiting the external validity of our findings for individuals with problematic alcohol use. Additionally, underreporting of alcohol consumption cannot be ruled out, potentially introducing reporting bias and affecting the internal validity of our results. We have now addressed these concerns explicitly in the limitations section.

Revised version:

In Discussion, lines 513-518: “Selection bias is a further consideration both from the initial participant recruitment and from missing data, as only 282 of the 345 enrolled participants had complete IFIS and 6-minute walk test results. Moreover, individuals with alcohol use disorder or high alcohol consumption were excluded based on screening questionnaires (2.3%), which may limit generalizability to this subgroup or reflect potential underreporting, given that this prevalence is unusually low for a Swedish population.”

**3.10 How many were invited to participate in ESOMIP and what was the acceptance rate of the initial study?**

Authors:

We acknowledge the reviewer’s concern regarding the absence of the total number of invited polulation and the acceptance rate. Patients with T2DM in Östergötland County attending annual checkups at their primary healthcare center between 2019 and 2023 were eligible for inclusion and were invited to participate by their diabetes nurse or treating physician. Due to the coronavirus disease 2019 pandemic and associated slow recruitment, additional invitations were sent via letters to patients registered at primary healthcare centers.

Given the large number of healthcare centers involved and the dual recruitment approach, it was not feasible to maintain a complete record of the total number of eligible individuals or calculate an accurate response rate. This dual recruitment approach has now been clarified in the Methods section. We also acknowledge that this limitation introduces the possibility of selection bias, which we have now explicitly stated in the Limitations section of the manuscript.

Revised version:

In Methods, lines 115-120: “Recruitment took place in Östergötland County, Sweden, primarily through four primary healthcare centers in the cities of Norrköping and Linköping. Individuals with T2DM were invited to participate during their routine annual check-ups with their diabetes nurse or treating physician. Due to slower enrolment during the coronavirus disease 2019 pandemic, additional invitations were sent by letter to patients registered at participating primary healthcare centers. The recruitment period spanned from 03/04//2019 to 22/09/2023.”

In Discussion, lines 513-518: “Selection bias is a further consideration both from the initial participant recruitment and from missing data, as only 282 of 345 eligible participants had complete IFIS and 6-minute walk test results. Moreover, individuals with alcohol use disorder or high alcohol consumption were excluded based on screening questionnaires (2.3%), which may limit generalizability to this subgroup or reflect potential underreporting, given that this prevalence is unusually low for a Swedish population.”

**3.11 Please clearly communicate if this was the primary endpoint of the ESOMIP-trial, a secondary endpoint, or an exploratory post hoc study. If this was a post hoc study, please also state that in the abstract.**

Authors:

Thank you, revised as suggested.

Revised version:

In Methods, line 113-114: “The present study focuses on an exploratory post hoc study within the EPSOMIP protocol.”

**3.12 Please explain the IFIS questionnaire in greater detail in methods. Time to complete, specifically trained staff or not, number of questions/points in total, established cutoffs or continuous scale. You validate two estimates in the study, how are they derived from the form?**

Authors:

As suggested, the paragraph has been corrected. Thanks.

Revised version:
In Methods, lines 149-161: “ Self-reported fitness was evaluated using the IFIS questionnaire, a brief, self-administered questionnaire originally validated in adolescents.[9] It takes approximately 1–2 minutes to complete and does not require trained personnel. The IFIS consists of five single-item questions, each addressing a different domain of perceived physical fitness: overall fitness, cardiorespiratory fitness, muscular fitness, speed-agility, and flexibility. Participants rate their fitness in each domain compared to peers of the same age, using a 5-point Likert scale: very poor (1), poor (2), average (3), good (4), and very good (5). This results in five separate scores, each ranging from 1 to 5, where higher scores indicate better perceived fitness in the respective domain. No established clinical cutoffs exist for the IFIS. For this study, the Swedish version of the IFIS questionnaire was used (available at: https://profith.ugr.es/ifis) with minor modifications to adapt the language for an adult population. The Swedish version has been validated in women during early pregnancy,[10] but not, to our knowledge, in adults with T2DM, the specific gap this study explores.”

**3.13 ”Thus, the IFIS provides an accessible measure that may be easily integrated into clinical practice for physical fitness or cardiometabolic risk assessment in patients with an increased risk of CVD, such as individuals with T2DM.” Please exemplify when this would be meaningful in order to promote a direct effect on T2D treatment outcomes, based on current evidence and current T2D treatment guidelines from eg. ADA. If this is not possible, please abstain from using the terminology ”clinical practice”.**

Authors:

Thanks for the comment. Although the ADA does not recommend a specific fitness assessment tool like IFIS, they do encourage assessing activity levels and functional capacity to guide individualized exercise prescriptions. In the statement about Comprehensive Medical Evaluation and Assessment of Comorbidities: Standards of Care in Diabetes-2025, the ADA states that “Ongoing management should be guided by the assessment of overall health and **functional status**, diabetes complications, cardiovascular risk, hypoglycemia risk, and shared decision-making to set therapeutic goals. Grade recommendation B” Including a validated instrument like IFIS can operationalize this recommendation by offering a structured, quick way to screen for low fitness, thus enabling targeted intervention to improve meaningful outcomes.

Revised version:

In Conclusions, lines 542-548: “For example, current American Diabetes Association (ADA) Standards of Care recommend assessing physical activity and fitness as part of a comprehensive lifestyle management strategy, given their close linked to improved glycemic control, reduced cardiovascular risk, and better treatment adherence.[47,48] While IFIS is not specifically endorsed by the ADA, incorporating brief, validated tools such as the IFIS could help identify patients with low fitness levels who may benefit from targeted physical activity interventions, potentially improving T2DM outcomes.”

**3.14 The contribution from the study participants should be acknowledged in acknowledgments.**

Authors:

Thank you, revised as suggested.

Revised version:

In Acknowledgments, lines 550-551: “We gratefully acknowledge the EPSOMIP participants for their time and valuable contribution to the study.”

**3.15 The ROC curve AUC are generally low in this study (clinically relevant AUC are generally at least >95% in order to be clinically useful). This is OK as you only use them for relative comparisons between the IFIS and walking test. However, this should be clearly stated so that readers get a full understanding of the results.**

Authors:

Thank you for this important comment. In response, we have calculated additional AUCs and diagnostic estimates (e.g., sensitivity, specificity) to further compare the IFIS with the 6-minute walk test. While the IFIS demonstrates acceptable criterion and construct validity, we acknowledge that its AUCs generally indicate poor to, at best, moderate discriminative ability. This limitation is now clearly addressed in the discussion, conclusion, and abstract of the main manuscript.

Revised version:

In Abstract, lines 53-58: “Conclusion: This study supports the International Fitness Scale (IFIS) may be a practical tool for adults with T2DM, showing evidence of criterion validity via graded association with 6MWT performance, and construct validity via significant associations with cardiometabolic and body composition outcomes. Although its ability to discriminate impaired functional capacity was limited, IFIS may serve as a feasible alternative for fitness assessment when objective testing is not possible in primary care.”

Please, see also revision version of comment 3.1.

**3.16 It is difficult from the results to understand how you can use statements as ”good”, ”strong” and so on. If similar terms are to be used. Please use a methological reference which supports the statement that this particular effect size is generally considered good or strong. Otherwise, please soften up these statements.**

Authors:

Thank you for the comment. As suggested, we have supported adjectives with objective criteria where possible and have harmonized or softened language throughout the manuscript—typically using terms such as “adequate” validity instead of “good” or “strong.” Additionally, the limited discriminative ability of the IFIS, as reflected by poor to moderate AUCs, has been clearly acknowledged in the discussion, conclusion, and abstract.

Revised version:

Please, see also mayor comments 3.1, 3.4 and 3.5.

**3.17 Why are only 4 of the 8 variables in life’s essential eight used? I do not find support for this in the reference used. What is the purpose of selecting these parameters instead of the more diabetes-relevant measures HbA1c, blood pressure, LDL and BMI which are actually clinically used for all patients with T2D.**

Authors:

Thanks for the relevant comment. The Life’s Essential (LE8) was developed by the American Heart Association (AHA) to evaluate overall cardiovascular health, shifting the focus from disease presence to a broader, more comprehensive health construct. Unlike isolated biomarkers such as HbA1c, LE8 incorporates both metabolic control and medication use, enabling a more accurate assessment of cardiovascular health. In our study, we focused specifically on LE8 factors (BMI, blood lipids, blood pressure, and diabetes) rather than behaviors (diet, sleep, physical activity, and smoking), as factors more directly reflect cardiometabolic outcomes. Importantly, because physical activity is part of LE8 behaviors and is conceptually and functionally related to IFIS, including it could introduce collinearity or overestimate associations. Therefore, we excluded behaviors to avoid bias and better isolate the relationship between fitness and cardiometabolic risk. This aspect has been clarified in Methods. Also, for clarity and to address a Reviewer’s request, the calculation of the LE8 factor score has been included in Supporting Information as Appendix 1.

Revised version:

In Methods, lines 180-188: “Cardiovascular health was assessed using the Life’s Essential 8 score, as defined by the American Heart Association.[22] In the present study, we focused specifically on Life’s Essential 8 health factors (BMI, blood lipids, blood pressure, and diabetes) rather than Life’s Essential 8 health behaviors (diet, sleep, physical activity, and smoking), as factors more directly reflect cardiometabolic outcomes. The Life´s Essential 8 Factor score accounts for actual values of non-HDL cholesterol, blood pressure, and blood glucose, along with the use of medication for these factors, as well as values of BMI. A detailed description of the scoring methodology has been provided as Supporting Information (Appendix 1).”

In Supporting Information, Appendix 1.

**3.18 As you z-transform most variables, you examine how the variability of the variables associate with IFIS, while information on the absolute effect on the outcomes can not be evaluated. Please use a language which clearly communicates this.**

Authors:

Thanks for the comment. A distinction has been made when we are referring to Z-scores in Results and in the titles of Figures S3, S4 and S5, and Tables S3 and S4.

**Reviewer #4:**

**Dear Dr. Herráiz-Adillo et al.,**

**Your study provides valuable evidence supporting IFIS as a practical tool for fitness assessment in T2DM. However, revisions are needed:**

Authors, general comment:

We would like to sincerely thank the reviewer for their thoughtful and constructive comments, which have helped improve the clarity and quality of our manuscript. As the manuscript is already quite lengthy, we have aimed to incorporate revisions as concisely as possible, while still ensuring that all key points are adequately addressed. We hope that our responses and the associated changes meet the reviewer’s expectations.

**4.1 Expand Limitations: Discuss the impact of cross-sectional design, unmeasured confounders (e.g., diet, socioeconomic status), and the 6-minute walk test’s suitability as a comparator.**

Authors:

Thanks for the relevant comment. In the context of a validation study, especially one focused on assessing criterion validity (e.g., how well the IFIS correlates with other measures of fitness), causal inference is not typically the goal, making a cross sectional study design appropriate. In fact, in STARD (STAndards for the Reporting of Diagnostic Accuracy Studies), one important criterion for assessing study quality and risk of bias is the temporal proximity between the index test and the reference standard. Thus, mentioning causality and confounding in this setting can be misleading. This aspect, along with other Reviewers’ comments, has now been clarified in the limitations section.

Revised version:

In Discussion, lines 495-502: “Several limitations should be acknowledged. Although the cross-sectional design is appropriate for validation studies, enabling simultaneous assessment of the index test (IFIS) and reference standards, it inherently limits causal inference. Additionally, unmeasured confounders such as T2DM duration and treatment, diet, physical activity, socioeconomic status, and comorbidities may have influenced the observed associations. However, our objective was not to infer causality but rather to assess how well IFIS scores align with the 6-minute walk test and established cardiometabolic and body composition markers, supporting its construct and criterion validity in individuals with T2DM.”

In Discussion, lines 519-522: “While the 6-minute walk test is a validated and practical field measure, it may not fully capture maximal cardiorespiratory capacity as laboratory-based assessments do, and may be influenced by varying motivation levels. Nonetheless, sensitivity analyses excluding low-effort performances yielded consistent results, supporting the robustness of our findings.”

**4.2 Reliability Data: Report test-retest reliability of IFIS in your cohort or explicitly state this as a study limitation.**

Authors:

Thank you for this important comment. Unfortunately, test-retest reliability was not assessed for the IFIS in our study. This limitation has already been acknowledged in the manuscript. To accommodate additional Reviewers’ comments, the paragraph about this limitation has been slightly modified.

Revised version:

In discussion, lines 503-508: “Test-retest reliability of IFIS was not assessed, though previous studies have demonstrated moderate to substantial reliability across populations,[9,10,45] with minimal influence of learning or fatigue effects. Nonetheless, some heterogeneity in the reliability of the IFIS across populations must be acknowledged.[46]”

**4.3 Clinical Utility: Acknowledge that IFIS AUCs (0.63–0.68) indicate "moderate" discrimination—useful for screening but insufficient for definitive diagnosis.**

Authors:

Thank you for the comment. This issue has now been extensively addressed to incorporate additional Reviewers’ comments. In Supporting information, Table S3, we have included the Diagnostic performance of the IFIS cardiorespiratory fitness test in identifying different levels of functional capacity as defined by the 6-minute walk test. In it, AUCs are in general poor-moderate, and this limited discriminative ability has been incorporated in the conclusions of the manuscript and abstract, as well as in the discussion.

Revised version:

In abstract, lines 53-58: “Conclusion: This study supports the International Fitness Scale (IFIS) may be a practical tool for adults with T2DM, showing evidence of criterion validity via graded association with 6MWT performance, and construct validity via significant associations with cardiometabolic and body composition outcomes. Although its ability to discriminate impaired functional capacity was limited, IFIS may serve as a feasible alternative for fitness assessment when objective testing is not possible in primary care.”

In Statistical analysis, lines 234-244: “To assess the diagnostic performance of the IFIS cardiorespiratory fitness item, we evaluated sensitivity, specificity, likelihood ratios, diagnostic odds ratios, and predictive values. Two thresholds were used to define reduced 6MWT performance:

i) a population-based threshold, defined as performance below the population-predicted mean 6-minute walk test distance (522.9 meters), calculated using the Enright & Sherrill equations based on the weighted average of age, height, and weight for men and women in the study population; and ii) an individualized threshold, also based on the Enright & Sherrill equations, which account for each participant’s age, sex, height, and weight. Functional impairment was defined as walking less than 80% of the individualized predicted distance.[17] Areas under the receiver operating characteristic (ROC) curves (AUCs) were calculated based on these thresholds to provide complementary insights into functional capacity.”

In Results, lines 313-319: “To identify individuals with reduced 6-minute walk test performance, defined as either walking below the population-predicted mean on the 6-minute walk test or achieving less than 80% of their predicted distance, reporting “very poor”, “poor” or “average” cardiorespiratory fitness on the IFIS showed a sensitivity of 93.9% (95% CI: 87.8, 97.5) and specificity of 19.0% (95% CI: 13.4, 25.8) for the former, and a sensitivity of 87.5% (95% CI: 61.7, 98.4) and specificity of 13.3% (95% CI: 9.4, 18.1) for the latter. The corresponding AUCs were 0.615 (95% CI: 0.552, 0.678) and 0.556 (95% CI: 0.404–0.708), respectively (see S3 Table).”

In Discussion, lines 421-440: “Moreover, reporting less than “good” cardiorespiratory fitness (i.e., “very poor,” “poor,” or “average”) on the IFIS yielded sensitivities of 93.9% and 87.5%, and negative predictive values of 82.1% and 94.4% for identifying individuals with reduced performance on the 6-minute walk test, defined as walking below the median distance or achieving less than 80% of their individualized predicted distance, respectively. Although the AUCs indicated poor discriminative ability (approximately 0.600), this may reflect the ordinal nature of IFIS, participants’ tendency to avoid selecting the extreme categories (“very poor” or “very good”), and the considerable variation in 6-minute walk test distances among participants within the same IFIS category, rather than a true lack of association. The 6-minute walk test is also not a formal gold-standard test, which should be considered when interpreting these results. Nevertheless, the high sensitivity and negative predictive values suggest that IFIS may serve as a practical first-line screening tool to flag individuals for further assessment. To our knowledge, this is the first study to evaluate the discriminative performance of IFIS using ROC curves in adults with T2DM, providing clinically interpretable metrics for identifying individuals with reduced functional capacity. These findings support the criterion validity of the IFIS cardiorespiratory item while highlighting its limitations as a standalone diagnostic measure. Future studies incorporating ROC analyses in independent cohorts are warranted to refine the discriminative performance of IFIS, improve its generalizability, and explore strategies to enhance the resolution of self-reported fitness measures.”

In Conclusion, lines 538-541: “While the IFIS showed limited ability to discriminate reduced functional capacity, it nonetheless offers an accessible and practical means of evaluating physical fitness and cardiometabolic risk in adults with T2DM. This is particularly relevant in settings such as primary care, where objective fitness assessments may be difficult to implement.”

**4.4 Generalizability: Address the homogeneity of your cohort and its implications for global applicability.**

Authors:

Thank you. A comment addressing the generalizability of our findings—also in response to related comments from other Reviewers—has now been incorporated into the manuscript.

Revised version:

In Discussion, lines 491-494: “Furthermore, the EPSOMIP cohort was drawn from unselected primary care patients with T2DM, including individuals across a wide range of ages, glycemic control levels, and comorbidity profiles. This enhances the external validity of our findings, making them generalizable to the broader population of adults with T2DM seen in routine clinical care.”

In Discussion, lines 513-518: “Selection bias is a further consideration both from the initial participant recruitment and from missing data, as only 282 of 345 eligible participants had complete IFIS and 6-minute walk test results. Moreover, individuals with alcohol use disorder or high alcohol consumption were excluded based on screening questionnaires (2.3%), which may limit generalizability to this subgroup or reflect potential underreporting, given that this prevalence is unusually low for a Swedish population.”

**4.5 Data Availability: Adhere to PLOS ONE policy by depositing data in a public repository or justifying restrictions ethically/legally. After revisions, this work will be a significant contribution to diabetes management.**

Authors:

Thanks a lot for reviewing the manuscript and for these kind words about the contribution of our paper.

Revised version:

“The data underlying this article cannot be made publicly available due to legal restrictions and the need to protect participant privacy. However, information on how to request access to the data in accordance with the guidelines of the Swedish Ethical Review Authority (email: registrator@etikprovning.se) can be obtained by contacting the study organization, Region Östergötland (email: region@regionostergotland.se; contact person: Mattias Ekstedt).”
