# Supplementary material for: Validity of the International Fitness Scale (IFIS) and its associations with cardiometabolic health and body composition in adults with type 2 diabetes: A cross-sectional study
Source: PLoS One. 2026 Jan 6;21(1):e0339364. doi: 10.1371/journal.pone.0339364 (PMC12774367; doi:10.1371/journal.pone.0339364)
Supplement: Attachment — Submitted filename: Response to reviewers R2_final.docx [file pone.0339364.s017.docx]

Dear Reviewers,

We are pleased to submit our revision to the original manuscript, PONE-D-25-22142R2

“Validity of the International Fitness Scale (IFIS) and Its Associations With Cardiometabolic Health and Body Composition in Adults With Type 2 Diabetes: A Cross-Sectional Study”

We would like to sincerely thank the reviewers for their thoughtful and constructive feedback. We hope that our responses and the corresponding changes meet the reviewers’ and the journal’s expectations.

An itemized, point-by-point response to the reviewers’ comments is provided in the Response to Reviewers file. The line numbers refer to the track-changes version of the manuscript, in which modifications are highlighted in yellow and deleted paragraphs are marked in red.

Sincerely,

Linköping, Sweden, December 03, 2025

Ángel Herraiz-Adillo, Postdoctoral Researcher, Linköping University, Sweden

Pontus Henriksson, Senior Associate Professor, Linköping University, Sweden

**Journal Requirements:**

**If the reviewer comments include a recommendation to cite specific previously published works, please review and evaluate these publications to determine whether they are relevant and should be cited. There is no requirement to cite these works unless the editor has indicated otherwise.**

**Please review your reference list to ensure that it is complete and correct. If you have cited papers that have been retracted, please include the rationale for doing so in the manuscript text, or remove these references and replace them with relevant current references. Any changes to the reference list should be mentioned in the rebuttal letter that accompanies your revised manuscript. If you need to cite a retracted article, indicate the article’s retracted status in the References list and also include a citation and full reference for the retraction notice.**

**Authors:**
We confirm that the reference list contains no retracted manuscripts and that no modifications to the references have been incorporated in the current revision. However, the reference list has been reordered to reflect the changes made in the manuscript.

**Additional Editor Comments:**
**The authors did not fully respond to one of the reviewers' comments regarding the article title: ''The title does not specify the study design as cross-sectional, which may mislead readers about the nature of the research, particularly since validity and associations could imply a longitudinal or experimental approach''**

**Authors:**

Thank you. In the previous revision, the article title was updated to ‘Validity of the International Fitness Scale (IFIS) and Its Associations With Cardiometabolic Health and Body Composition in Adults With Type 2 Diabetes: A Cross-Sectional Study’, which explicitly indicates the cross-sectional nature of the work. However, we would be happy to revise the title further if the editor considers it necessary.

**Review Comments to the Author**

**Reviewer #3: All comments addressed to satisfaction. I have no additional comments. All my prints have good answers and revisions met my feedback**

**Authors:**

Thank you for the constructive comments.

**Reviewer #5:**

**Dear Authors,**

**Thank you for the opportunity to review this manuscript. I am attaching some suggestions for improving the initial version of your investigation:**

**Abstract**

**Lines 31-60:**

**- The structure of the abstract appropriately highlights the objectives, methodology, and results of the research;**

**- The structure of the abstract highlights the issues of the topic addressed, also mentioning the main objective of the research.**

**Authors:**

Thank you for the appreciation of our work.

**Introduction**

**Therefore, the context and background of the research provides information about the field of study, the problems existing in it, and the progress made previously.**

**Lines 97-105 - In this part, it is recommended that, in addition to the objectives, the purpose of the study and the research hypotheses be specified.**

**Authors:**

Thank you for the comments. We have added the research hypotheses as suggested. Considering that the manuscript is already very long (> 5000 words) after revising the manuscript according to the comments in the first round of revisions, we suggest not adding a separate section on the study’s purpose, as it is already implicit in the aim and hypotheses. We remain open to further adjustments if requested by the editor.

**Revised version:**

In Introduction, lines 97-98:

“We hypothesized that the IFIS is a valid tool for assessing physical fitness in adults at high cardiovascular risk, particularly those with T2DM. Thus, the aims of this study were: 1) to investigate the validity of the IFIS self-reported physical fitness scores (i.e., overall and cardiorespiratory fitness) in assessing cardiorespiratory fitness considering the objectively measured 6-minute walk test as reference (criterion validity), and 2) to examine and compare the associations of the IFIS self-reported physical fitness scores and the 6-minute walk test with a wide range of cardiometabolic and body composition outcomes in adults with T2DM (construct validity).”

**Methods**

**In the section – methods – the purpose is described clearly, precisely and completely. Essential information is included.**

**Lines 134-139 - the study diagram illustrates the sequence of research stages and the relationships between them, providing a clear and structured image of the work process.**

**Authors:**

Thank you very much.

**Discussion**

**- In this section, the results obtained in the study are contextualized, being detailed as theoretical and practical implications based on the relevant results of the present study. The limitations of the study and the strengths identified in the study are described in accordance with the relevance of the study.**

**Authors:**

Thank you for your appreciation.

**Conclusions**

**Lines 525-541 – it is recommended to rewrite the conclusions because they are too extensive and some of them can be moved to the practical implications. To reformulate future research directions in correlation with the relevant results of the study.**

**Authors:**

Thank you for this helpful comment. As recommended, we have rewritten the Conclusions to make them more concise and focused. We have also reformulated the future research directions to align them more clearly with the results obtained in our analysis.

**Revised version:**

In Discussion, lines 479-487:

“In this sense, while the IFIS showed limited ability to discriminate reduced functional capacity, it demonstrates acceptable criterion and construct validity, and remains a highly feasible option, requiring only a single self-reported item that can be completed in 1–2 minutes.[10] This is especially important in primary care settings managing patients with T2DM, where objective fitness assessments are often difficult to implement. In line with this, the current American Diabetes Association Standards of Care recommend evaluating physical activity and fitness as part of comprehensive lifestyle management, given their association with improved glycemic control, reduced cardiovascular risk, and enhanced treatment adherence.[41,42]”

In Discussion, lines 528-533:
“Further research is needed to assess the longitudinal validity of the IFIS and its predictive value for clinically relevant hard outcomes, including all-cause and CVD-related mortality and morbidity. Validation in independent T2DM cohorts is also necessary to confirm the generalizability of the ROC-based performance observed here. In addition, evaluating test–retest reliability and comparing IFIS with broader objective fitness measures would help determine its suitability for routine monitoring and clinical decision-making.”

In Conclusions, lines 535-553:

“Our findings extend previous research by demonstrating that the IFIS is a valid and feasible tool for assessing physical fitness in adults not only at low risk of CVD,[9,10,38,45–48] but also at high risk, such as adults with T2DM. While the IFIS showed limited ability to discriminate reduced functional capacity, it nonetheless offers a practical tool for evaluating physical fitness in adults with T2DM. This is particularly relevant in settings such as primary care, where objective fitness assessments may be difficult to implement. Validated tools such as the IFIS could help identify patients with low fitness levels who may benefit from targeted physical activity interventions, potentially improving T2DM outcomes.”

**Reviewer #6: All comments have been addressed properly, but consider and investigate belows vague ones:**

**Reviewer 2 question: The title does not specify the study design as cross-sectional, which may mislead readers about the nature of the research, particularly since validity and associations could imply longitudinal or experimental approach.**

**I think the authors do not respond this one correctly.**

**Authors:**

Thank you. As we commented previously, we updated the article title to ‘Validity of the International Fitness Scale (IFIS) and Its Associations With Cardiometabolic Health and Body Composition in Adults With Type 2 Diabetes: A Cross-Sectional Study’, which explicitly indicates the cross-sectional nature of the work. However, we would be happy to revise the title further if the editor considers it necessary.
